# Unraveling the ameliorative effects of Ling-Gui-Zhu-Gan decoction on MASLD: an integrative gut microbiota and lipidomic study

**DOI:** 10.3389/fmicb.2026.1916850

**Published:** 2026-09-03

**Authors:** Juanhong Lei, Dihong Gong, Yaqin Yang, Li Li, Yue Zhou, Guanrong Qiao, Xingxin Yang, Jie Yu

**Affiliations:** 1 The First Clinical College, Yunnan University of Chinese Medicine, Kunming, China; 2 School of Chinese Materia Medica and Yunnan Key Laboratory of Southern Medicine Utilization, Yunnan University of Chinese Medicine, Kunming, China

**Keywords:** gut microbiota, gut-liver axis, Ling-Gui-Zhu-Gan decoction, lipidomics, steatotic liver disease

## Abstract

**Background:**

The prevalence of metabolic dysfunction-associated steatotic liver disease (MASLD) has been continuously increasing. However, the pathogenesis of MASLD is quite complex and involves multiple factors, leading to a lack of targeted therapeutic drugs for MASLD. Ling-Gui-Zhu-Gan Decoction (LGZGD) has demonstrated significant hepatoprotective and lipid-lowering effects, yet its mechanism of action against MASLD remains incompletely understood.

**Methods:**

In this study, we established a high-fat diet-induced MASLD rat model to investigate the pharmacological effects of LGZGD on MASLD from the perspectives of gut microbiota and lipidomics.

**Results:**

Using UPLC-Q-TOF-MS analysis, we putatively identified 83 chemical components in LGZGD, primarily flavones and terpenoids, which may represent the key active constituents responsible for its ameliorative effects. In MASLD model rats, LGZGD treatment reduced the levels of TC, TG, AST, ALT, and LDL-C in both serum and liver tissue, while increasing HDL-C levels. Analysis of the gut microbiota revealed that LGZGD treatment increased the abundance of *g__Collinsella* and *g__Prevotellaceae_UCG-003*, and decreased the abundance of *g__Faecalibaculum*. Furthermore, LGZGD was found to regulate 831 lipids, primarily involved in metabolic pathways such as glycerophospholipid, glycerolipid, and sphingolipid metabolism.

**Conclusions:**

This study demonstrates that LGZGD prevents MASLD, and its mechanism of action involves the regulation of gut microbiota and the modulation of lipid metabolism.

## Graphical Abstract

**Figure:**
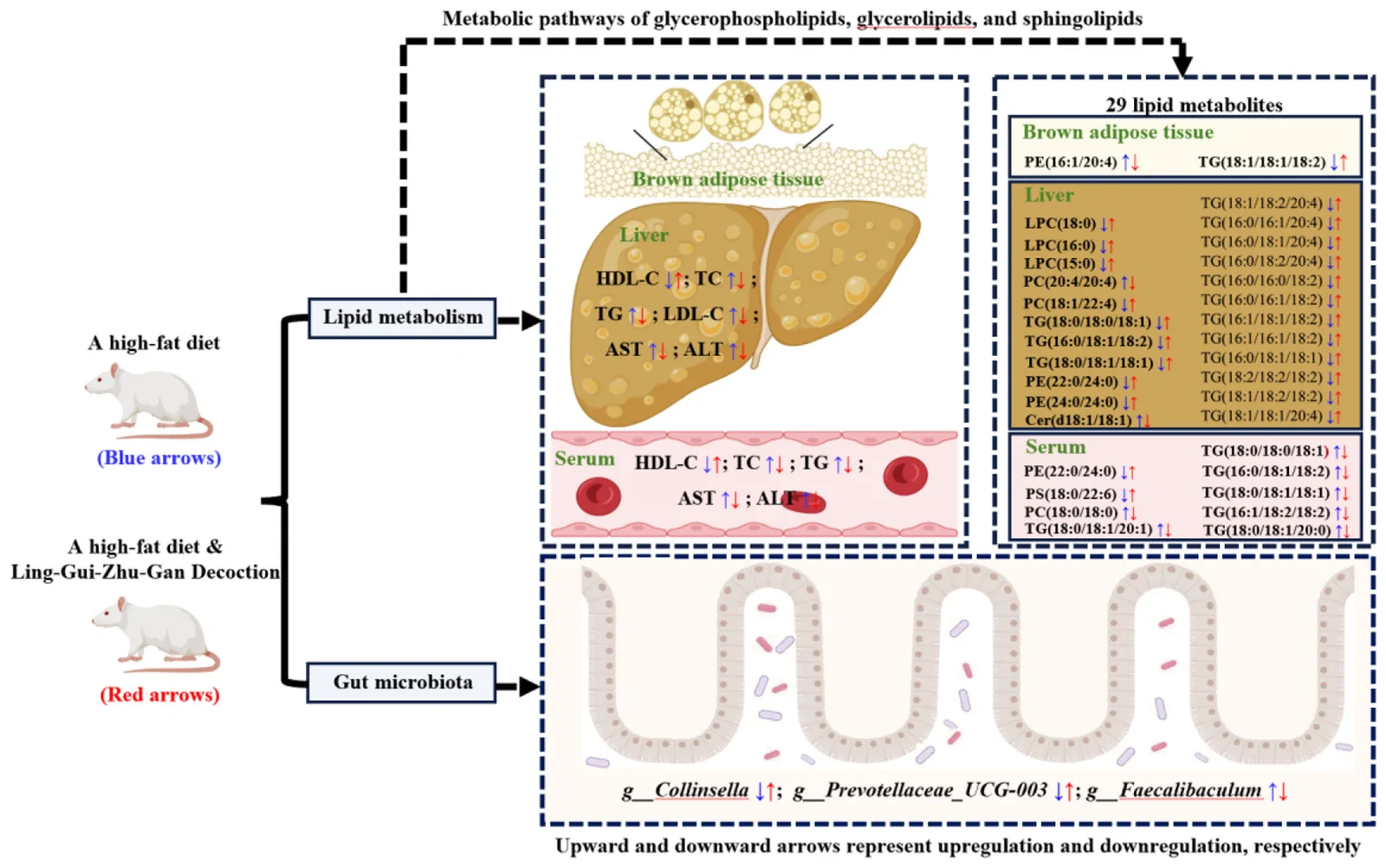
LGZGD ameliorates MASLD through modulation of gut microbiota and lipid metabolism.

## Highlights

- LGZGD contains multiple constituents, such as flavones and terpenoids
- LGZGD prevents dysbiosis to relief MASLD
- LGZGD restores 831 lipid species, primarily involved in three metabolic pathways

## 1 Introduction

Metabolic dysfunction-associated steatotic liver disease (MASLD), previously termed non-alcoholic fatty liver disease (NAFLD), is defined as hepatic steatosis accompanied by metabolic dysregulation associated with overweight/obesity, type 2 diabetes mellitus, or other metabolic abnormalities (Xue and Fan, 2020). As one of the most prevalent chronic liver diseases globally, the prevalence of MASLD continues to rise, with a noticeable trend toward affecting younger populations, making the search for targeted therapies increasingly urgent. However, the pathogenesis of MASLD is highly complex, involving multiple factors such as oxidative stress, inflammation, lipotoxicity, mitochondrial dysfunction, and gut microbiota dysbiosis (Kuchay et al., 2020; Shi et al., 2026; Wang et al., 2025; Yin et al., 2026). Consequently, there remains a notable paucity of effective targeted interventions for MASLD. Although numerous agents have been investigated, developing safe and efficacious options continues to pose a significant challenge.

Although the term “fatty liver” does not appear in traditional Chinese medicine (TCM), certain herbal formulas have been historically used for liver-related disorders. Notably, the Ling-Gui-Zhu-Gan Decoction (LGZGD) has attracted attention due to its potential metabolic benefits. The LGZGD was first documented in “*Synopsis of the Golden Chamber*” (Jin Gui Yao Lue) (Zhang and Wang, 1994) and is recognized as the representative formula of the “Linggui category.” It is composed of four herbal medicines: *Poria* (PR), *Cinnamomi Ramulus* (CR), *Atractylodis Macrocephalae Rhizoma* (AM), and *Glycyrrhizae Radix et Rhizoma* (GR) (For comprehensive details, refer to Supplementary Table S1). Modern pharmacological studies have provided evidence supporting the ameliorative potential of LGZGD in MASLD. Previous *in vivo* studies have shown that LGZGD treatment significantly reduces hepatic steatosis, lowers serum triglyceride and cholesterol levels, and improves insulin sensitivity in high-fat diet (HFD)-induced rodent models of MASLD (Dang et al., 2019; Ning et al., 2022; Wu et al., 2019; Xu et al., 2026; Zhang et al., 2017; Zhu et al., 2017). *In vitro* experiments using hepatocyte cell lines have demonstrated that LGZGD-containing serum or its active components inhibit intracellular lipid accumulation and reduce the production of pro-inflammatory cytokines (Cao et al., 2022; Wang et al., 2024). Furthermore, LGZGD has been reported to ameliorate oxidative stress by enhancing antioxidant enzyme activities in the liver of HFD-fed animals (Yang et al., 2017). These findings provide a strong scientific rationale for further investigating the effects of LGZGD in animal models of MASLD. However, while existing studies have investigated the pharmacological effects of this formula from multiple perspectives, there remains a gap in in-depth research exploring the mechanism of LGZGD in treating MASLD through the interplay between gut microbiota and lipid metabolism. Therefore, systematically elucidating the mechanism of LGZGD from an integrated microbiome-metabolome perspective will not only help reveal its holistic regulatory characteristics but also provide new insights and experimental evidence for the modern research of classical TCM prescriptions.

**Figure 1:**
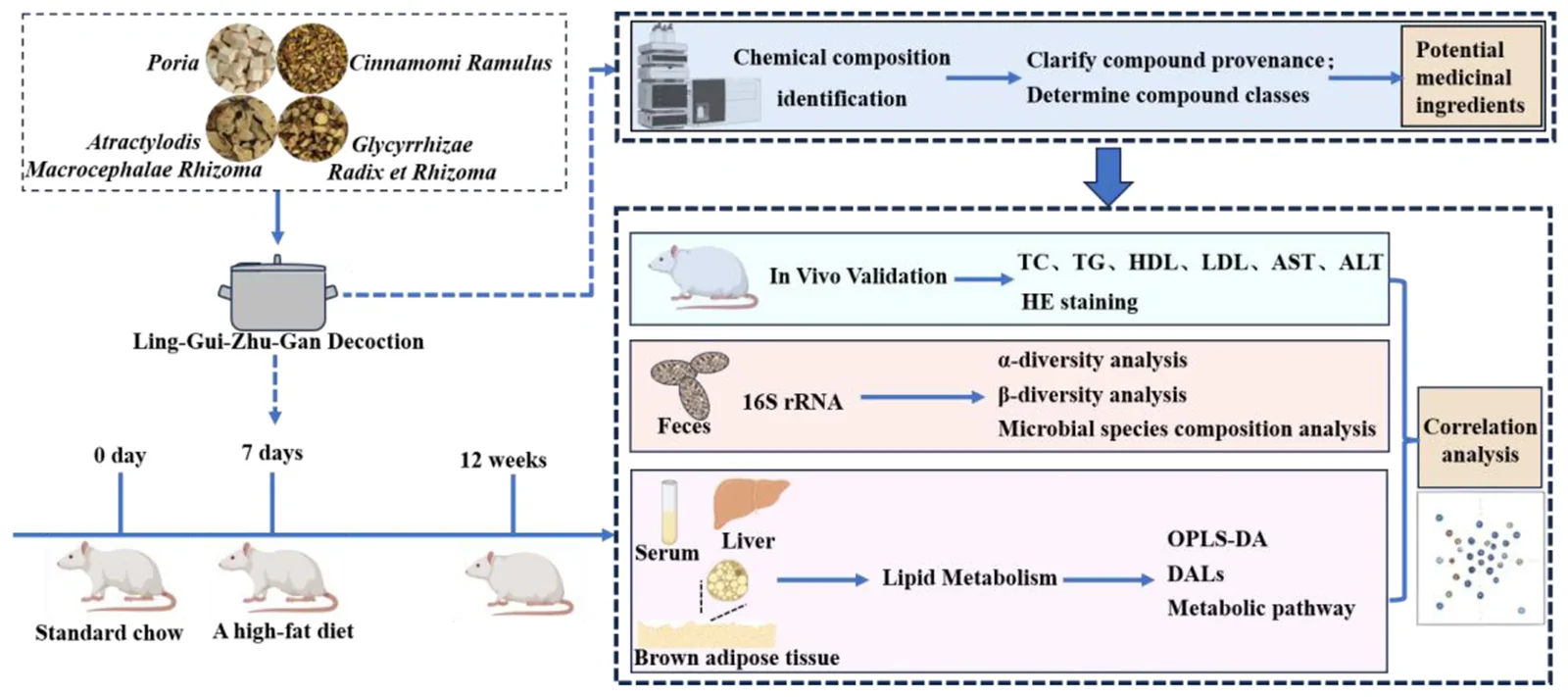
Research process.

The present study first conducted a comprehensive and in-depth analysis of the chemical constituents in LGZGD using ultrahigh performance liquid chromatography-four-stage pole-time-of-flight tandem mass spectrometry (UPLC-Q-TOF-MS). Subsequently, a rat model of MASLD was established to evaluate the pharmacological effects of LGZGD on MASLD. The 16S rRNA sequencing technique was employed to determine whether a high-fat diet (HFD) could induce gut microbiota dysbiosis, thereby verifying the regulatory effects of LGZGD on the gut microbiota. Finally, untargeted lipidomics was utilized to obtain lipid metabolic profiles of serum, liver, and brown adipose tissue (BAT), aiming to elucidate whether LGZGD could ameliorate MASLD by modulating lipid metabolism disorders (Figure 1). By integrating chemical composition analysis, *in vivo* pharmacological evaluation, gut microbiota assessment, inflammatory marker detection, and lipidomic profiling, this work aims to provide a comprehensive scientific basis for the application of LGZGD in managing MASLD, while also offering new insights into the mechanism of herbal medicine in regulating metabolic diseases.

## 2 Materials and methods

### 2.1 Preparation of LGZGD

PR (55.2 g, Cat. No. C240307005) and CR (41.4 g, Cat. No. C240318002) (both purchased from Yunnan Zongshun Biotechnology Co., Ltd., Kunming, China), as well as AM (41.4 g, Cat. No. 24071003) and GR (27.6 g, Cat. No. 24041902) (both purchased from Yunnan Zhanyi Pharmaceutical Co., Ltd., Kunming, China), were accurately weighed, mixed, and added to 1,200 mL of purified water. The mixture was soaked for 1 h, then boiled vigorously over high heat, followed by simmering over low heat until the decoction volume was reduced to 600 mL. The decoction was filtered and lyophilized to obtain LGZGD lyophilized powder. The extraction yield of the decoction was 17.50%.

### 2.2 Chemical composition identification

A 0.5 g aliquot of LGZGD freeze-dried powder was dissolved in 10 mL of methanol and ultrasonicated for 45 min. The solution was then centrifuged at 12,000 r/min for 10 min. The supernatant was filtered through a 0.22 μm microporous membrane, and the resulting filtrate was collected in an injection vial for UPLC-Q-TOF-MS analysis.

A 5 μL aliquot of the sample was separated on a Waters ACQUITY UPLC BEH C18 column (2.1 mm × 100 mm, 1.7 μm) prior to mass spectrometric detection. The mobile phase consisted of (A) 0.1% aqueous formic acid and (B) acetonitrile, with the following gradient elution program: 0–10 min, 5%−20% B; 10–15 min, 20%−23% B; 15–30 min, 23%−32% B; 30–40 min, 32%−33% B; 40–50 min, 33%−38% B; 50–60 min, 38%−48% B; 60–70 min, 48%−65% B; 70–75 min, 65%−95% B; 75–85 min, 95% B. The column temperature was maintained at 30 °C with a constant flow rate of 0.2 mL/min. The analysis was performed using an electrospray ionization (ESI) source operating in both positive and negative ion modes. The drying gas temperature is 320 °C, and the drying gas flow rate is 8 L/min. The sheath gas temperature is 350 °C, and the sheath gas flow rate is 11 L/min. The atomization gas pressure is 35 pis. The nozzle voltage is 3.5 KV, and the capillary voltage is 1,000 V. Scanning range: m/z 50–1,500; Collision energy: 30, 40. Atomizing gas, drying gas, and sheath gas are all high-purity nitrogen gas. The acquired mass spectral data were compared with reference standards and published MS data. Compounds with mass accuracy errors (δ) less than 10 ppm (1 ppm = 1 × 10^−6^) were selected for structural identification of chemical constituents in the sample.

### 2.3 Animal study

Eight-week-old male Sprague-Dawley (SD) rats (body weight 180 ± 20 g) of specific pathogen-free (SPF) grade were supplied by Chengdu Zhili Experimental Animal Co., Ltd. (Chengdu, China; License No.: SCXK (Chuan) 2015-030). The animals were housed under standard laboratory conditions at the Animal Experiment Center of Yunnan University of Chinese Medicine. The experimental procedures were approved by the Institutional Animal Care and Use Committee of Yunnan University of Chinese Medicine (Ethical Approval No.: R-062021081).

After 1 week of acclimatization with standard diet, the rats were randomly assigned to five groups (*n* = 6 per group) as follows: normal control (NC) group, HFD group, fenofibrate (FC) group, low-dose LGZGD (LGZGD-L) group, and high-dose LGZGD (LGZGD-H) group. Based on the equivalent dose conversion between humans and rats according to body surface area, the clinical equivalent dose of FC was set at 20 mg/kg/d, and that of LGZGD was 14.9 g crude herb/kg/d. The latter was designated as the low dose in this experiment, while the high dose was set at four times the low dose, i.e., 59.6 g crude herb/kg/d. Given the extraction yield of 17.50% (lyophilized powder/crude herb), the administered doses of LGZGD powder were 2.61 g powder/kg/d for the low-dose group (equivalent to 14.9 g crude herb/kg/d) and 10.43 g powder/kg/d for the high-dose group (equivalent to 59.6 g crude herb/kg/d). The lyophilized powder was suspended in physiological saline to prepare a gavage solution at a concentration of 0.261 g powder/mL for the low-dose group and 1.043 g powder/mL for the high-dose group; all rats received a fixed gavage volume of 10 mL/kg body weight. All drug interventions were administered via oral gavage once daily. The NC group received standard chow, while the other groups were fed a HFD (79% standard chow + 10% lard + 10% egg yolk powder + 1% cholesterol) for 12 weeks to establish the MASLD model. Drug interventions were administered concurrently during this period. Body weight was measured once per week. At the end of the 12th week, following the final administration, fecal samples were collected from each rat via anal insertion using sterile EP tubes and immediately snap-frozen in liquid nitrogen.

After a 12-h fasting period, the rats were euthanized by sodium pentobarbital injection (100 mg/kg, i.p.), and then the liver, serum, perirenal white adipose tissue (PWAT), epididymal white adipose tissue (EWAT), and BAT were collected. Serum samples and portions of the tissue samples were stored at −80 °C for subsequent analysis.

### 2.4 Histological analysis

The left lateral lobe of rat liver was collected for histopathological analysis. Rat liver tissues were fixed in 10% neutral formalin and embedded in paraffin. The paraffin-embedded liver blocks were sectioned at 4 μm thickness and stained with hematoxylin and eosin (H&E) to visualize lipid accumulation patterns. Histological features were observed and imaged using a light microscope.

### 2.5 Biochemical analysis

Serum and liver tissue samples were analyzed using commercial assay kits (Nanjing Jiancheng Bioengineering Institute, Nanjing, China) in accordance with the manufacturer's instructions. The levels of alanine aminotransferase (ALT, Cat. No. C009-2-1), aspartate aminotransferase (AST, Cat. No. C010-2-1), triglycerides (TG, Cat. No. A110-1-1), total cholesterol (TC, Cat. No. A111-1-1), high-density lipoprotein cholesterol (HDL-C, Cat. No. A112-1-1), and low-density lipoprotein cholesterol (LDL-C, Cat. No. A113-1-1) in both serum and liver tissues were measured.

### 2.6 16S rRNA gene sequencing and bioinformatic analysis

Genomic DNA was extracted from the samples using the OMEGA Mag-bind Soil DNA Kit. The purity and concentration of the extracted DNA were measured. The V3–V4 hypervariable region of the bacterial 16S rRNA gene was amplified using the barcode-specific primer pair 341F (5′-CCTAYGGGRBGCASCAG-3′) and 806R (5′-GGACTACNNGGGTATCTAAT-3′) with a high-fidelity DNA polymerase. PCR was performed in a total volume of 30 μL, consisting of 15 μL of 2 × Phusion Master Mix, 3 μL of each primer (2 μM), 10 μL of genomic DNA (1 ng/μL, corresponding to 5–10 ng template), and 2 μL of nuclease-free water. The thermal cycling protocol comprised an initial denaturation at 98 °C for 1 min, followed by 30 cycles of 98 °C for 10 s, 50 °C for 30 s, and 72 °C for 30 s, with a final extension at 72 °C for 5 min. Library construction was performed with the TruSeq Nano DNA LT Library Prep Kit. The constructed libraries were quality-checked using the Agilent Bioanalyzer 2100 and Promega QuantiFluor system. Only qualified libraries were subjected to sequencing. After sequencing, parameters were configured as required to generate representative sequences and an ASV abundance table. Representative sequences for each ASV were selected using the QIIME 2 software package and subsequently aligned and annotated against a reference database. Rarefaction and rank-abundance curves were plotted. Alpha and beta diversity indices were calculated, and species composition was analyzed.

### 2.7 Lipidomic analysis

The serum sample processing procedure was as follows: A 200 μL aliquot of serum was pipetted into an Eppendorf tube, followed by the addition of 80 μL methanol and 400 μL methyl tert-butyl ether (MTBE). The mixture was vortexed for 30 s and subjected to ultrasonic extraction for 30 min (5 °C, 40 kHz). After incubation at −20 °C for 30 min, the sample was centrifuged at 13,000 × g for 15 min at 4 °C. Then, 350 μL of the supernatant was collected, dried in a vacuum concentrator, and reconstituted in 100 μL of reconstitution solution (isopropanol: acetonitrile = 1:1, v/v). The solution was vortexed for 30 s and sonicated in an ice-water bath for 5 min (40 kHz). Following centrifugation at 13,000 × g for 5 min at 4 °C, 80 μL of the supernatant was transferred into an injection vial equipped with an insert for subsequent instrumental analysis.

For tissue samples, approximately 50 mg of liver or BAT was accurately weighed and placed into a 2 mL centrifuge tube, each containing one 6 mm grinding bead. Then, 280 μL of extraction solvent (methanol: water = 2:5, v/v) and 400 μL of MTBE were added. Homogenization was performed using a cryogenic tissue grinder for 6 min (−10 °C, 50 Hz). The homogenate was treated with low-temperature ultrasonic extraction for 30 min (5 °C, 40 kHz) and incubated at −20 °C for 30 min. After centrifugation at 13,000 × g for 15 min at 4 °C, 350 μL of the supernatant was transferred to a new microcentrifuge tube and evaporated under a gentle nitrogen stream. The residue was reconstituted in 100 μL of reconstitution solution (isopropanol: acetonitrile = 1:1, v/v), vortexed for 30 s, and sonicated in an ice-water bath for 5 min (40 kHz). A final centrifugation step was performed at 13,000 × g for 10 min at 4 °C, after which the supernatant was transferred to an injection vial with an insert for analysis.

After instrumental analysis, the raw LC-MS data were imported into LipidSearch software (Thermo, CA, USA) for lipidomics processing, including baseline filtering, peak detection, integration, retention time correction, peak alignment, and identification. This generated a data matrix containing lipid names, retention times, mass-to-charge ratios (m/z), and peak intensities. Feature identification was performed using LipidSearch by matching MS and MS/MS spectra against metabolic databases. The mass error tolerance for MS was set to < 10 ppm, and metabolites were identified based on MS/MS spectral matching scores. The resulting matrix was uploaded to the Majorbio Cloud Platform for further analysis. First, data preprocessing was performed. The data matrix was processed using the 80% rule to remove missing values, meaning that only variables with non-zero values in at least 80% of the samples in one group were retained. Missing values were then filled with the minimum value from the original matrix. To minimize errors from sample preparation and instrument instability, the peak intensities were subjected to total sum normalization, yielding a normalized data matrix. Additionally, variables with a relative standard deviation (RSD) > 30% in the QC samples were removed. Finally, a log10 transformation was applied to obtain the final data matrix for subsequent analysis. Differential analysis was performed on the preprocessed data matrix. Orthogonal partial least squares-discriminant analysis (OPLS-DA) was conducted. In addition, Student's t-test and fold-change analysis were carried out. The selection of differential metabolites was performed based on the values of variable importance in the projection (VIP) from the OPLS-DA model and the *p*-values derived from Student's *t*-tests. Metabolites with VIP > 1 and *p* < 0.05 were considered statistically significant differential metabolites. These differential metabolites were subsequently annotated using the KEGG database to identify the metabolic pathways in which they are involved.

### 2.8 Correlation analysis

By analyzing the correlation between pharmacological indicators, genus level differential bacteria, and differential lipids and lipid metabolism pathways, network diagrams were drawn to clarify the mechanism of action of LGZGD.

### 2.9 Statistical analysis

Data are expressed as the mean ± standard error of the mean. Statistical analyses were conducted using GraphPad Prism version 7.0 (GraphPad Software, La Jolla, CA, USA). For multiple comparisons among groups, one-way analysis of variance (ANOVA) was used, followed by Tukey's *post-hoc* test when appropriate. For data that did not meet the assumptions of normality or homogeneity of variance, non-parametric tests, such as the Kruskal–Wallis test, were employed. A two-sided *p*-Value < 0.05 was considered statistically significant.

## 3 Results

### 3.1 Chemical constituents identified in LGZGD

The chemical constituents of LGZGD were analyzed using a UPLC-Q-TOF-MS system in both positive and negative ionization modes, yielding the base peak chromatograms (BPC, Supplementary Figure S1). A total of 83 compounds were putatively identified in LGZGD, including seven compounds derived from PR, three compounds from CR, eight compounds from AM, 60 compounds from GR, and five compounds shared by two or more medicinal herbs. Among the 83 compounds detected, 38 belong to flavones, 24 belong to terpenes, six belong to organic acids, three belong to phenylpropanoids, two belong to glycosides, and 10 belong to other types. For comprehensive details, refer to Supplementary Table S2.

### 3.2 LGZGD alleviated HFD-induced hepatic injury and lipid accumulation

**Figure 2:**
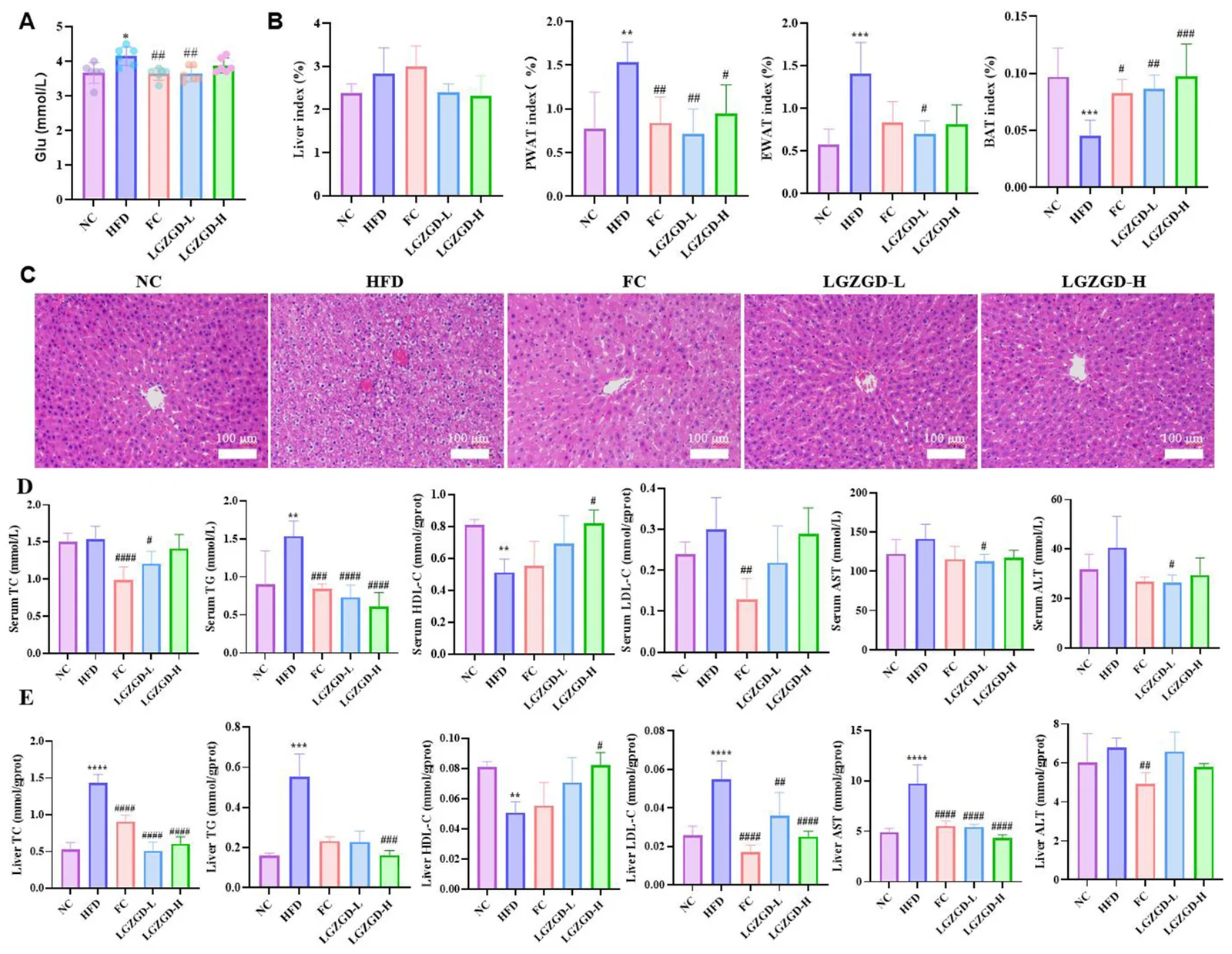
Effects of LGZGD on metabolic parameters and liver histopathology in HFD-fed rats. **(A)** FBG Levels. **(B)** Relative Weights of the Liver and Adipose Tissue. **(C)** Representative images of hematoxylin and eosin staining of liver sections. Scale bar: 100 μm. **(D)** The serum TC, TG, HDL-C, LDL-C, ALT and AST levels. **(E)** The Liver TC, TG, HDL-C, LDL-C, ALT and AST levels. (*n* = 6, *p < 0.05, **p < 0.01, ***p < 0.001, ****p < 0.0001 vs. NC group; ^#^*p* < 0.05, ^##^*p* < 0.01, ^###^*p* < 0.001, ^####^*p* < 0.0001 vs. HFD group). ALT, alanine aminotransferase; AST, aspartate aminotransferase; FBG, fasting blood glucose; HDL-C, high-density lipoprotein cholesterol; HFD, high-fat diet; LDL-C, low-density lipoprotein cholesterol; LGZGD, Ling-Gui-Zhu-Gan Decoction; NC, normal control; TC, total cholesterol; TG, triglycerides.

As shown in Figure 2A, compared with the NC group, the fasting blood glucose (FBG) level in the HFD group was significantly increased (*p* < 0.05). Compared with the HFD group, FBG levels in the FC and LGZGD-L groups were significantly decreased (*p* < 0.01), while the FBG level in the LGZGD-H group also showed a reduction, though without statistical significance. Organ indices [calculated as organ weight to body weight at sacrifice, expressed in grams per gram (g/g)] revealed that although the liver index in the HFD group exhibited an increasing trend relative to the NC group, the difference was not statistically significant; in contrast, the indices of subcutaneous white adipose tissue (PWAT) and epididymal white adipose tissue (EWAT) were significantly elevated, whereas the brown adipose tissue (BAT) index was significantly reduced. Compared with the HFD group, the FC group showed no significant changes in liver index or EWAT index, a significant decrease in PWAT index (*p* < 0.01), and a significant increase in BAT index (*p* < 0.05). Compared with the HFD group, the LGZGD-L group exhibited no significant changes in liver index, but PWAT and EWAT indices were significantly decreased (*p* < 0.01 and *p* < 0.05, respectively), and BAT index was significantly increased (*p* < 0.01); compared with the HFD group, the LGZGD-H group showed no significant changes in liver index or EWAT index, a significant decrease in PWAT index (*p* < 0.05), and a significant increase in BAT index (*p* < 0.001). Collectively, these results indicated that FC and LGZGD effectively inhibited HFD-induced elevation of blood glucose, alleviated hypertrophy of white adipose tissue, and markedly increased BAT index (Figure 2B).

Histopathological and biochemical analyses confirmed the hepatoprotective effects of FC and LGZGD. H&E-stained sections showed that the liver tissues in the FC and LGZGD groups exhibited clear and intact structural architecture with uniformly arranged hepatocytes, in stark contrast to the HFD group (Figure 2C). Regarding biochemical parameters, compared with the NC group, the HFD group had a significantly elevated serum TG level (*p* < 0.01), with non-significant increasing trends in TC, ALT, AST, and LDL-C levels. In addition, the HDL-C level was significantly decreased (*p* < 0.01). Compared with the HFD group, the FC group showed significant reductions in TC, TG, and LDL-C (*p* < 0.0001, *p* < 0.001, and *p* < 0.01, respectively), with no significant changes in HDL-C, AST, or ALT. Compared with the HFD group, the LGZGD-L group exhibited significant decreases in TC, TG, AST, and ALT (*p* < 0.05, *p* < 0.0001, *p* < 0.05, and *p* < 0.05, respectively), while HDL-C and LDL-C showed no significant changes; the LGZGD-H group showed a significant reduction in TG (*p* < 0.0001) and a significant increase in HDL-C (*p* < 0.05; Figure 2D). In liver tissue, compared with the NC group, the HFD group had significantly elevated levels of TC, TG, LDL-C, and AST (*p* < 0.0001, *p* < 0.001, *p* < 0.0001, and *p* < 0.0001, respectively), a non-significant increase in ALT, and a significant decrease in HDL-C (*p* < 0.01). Compared with the HFD group, the FC group showed significant reductions in TC, LDL-C, AST, and ALT (*p* < 0.0001, *p* < 0.0001, *p* < 0.0001, and *p* < 0.001, respectively), with no significant changes in TG or HDL-C. Compared with the HFD group, the LGZGD-L group exhibited significant decreases in TC, LDL-C, and AST (*p* < 0.0001, *p* < 0.05, and *p* < 0.0001, respectively), with no significant changes in TG, ALT, or HDL-C; the LGZGD-H group showed significant reductions in TC, TG, LDL-C, and AST (*p* < 0.0001, *p* < 0.001, *p* < 0.0001, and *p* < 0.0001, respectively), and a significant increase in HDL-C (*p* < 0.05; Figure 2E). These results suggest that LGZGD intervention effectively ameliorated dyslipidemia and hepatic injury, indicating its potential as a preventive intervention for MASLD.

### 3.3 LGZGD ameliorated HFD-induced gut microbiota dysbiosis

**Figure 3:**
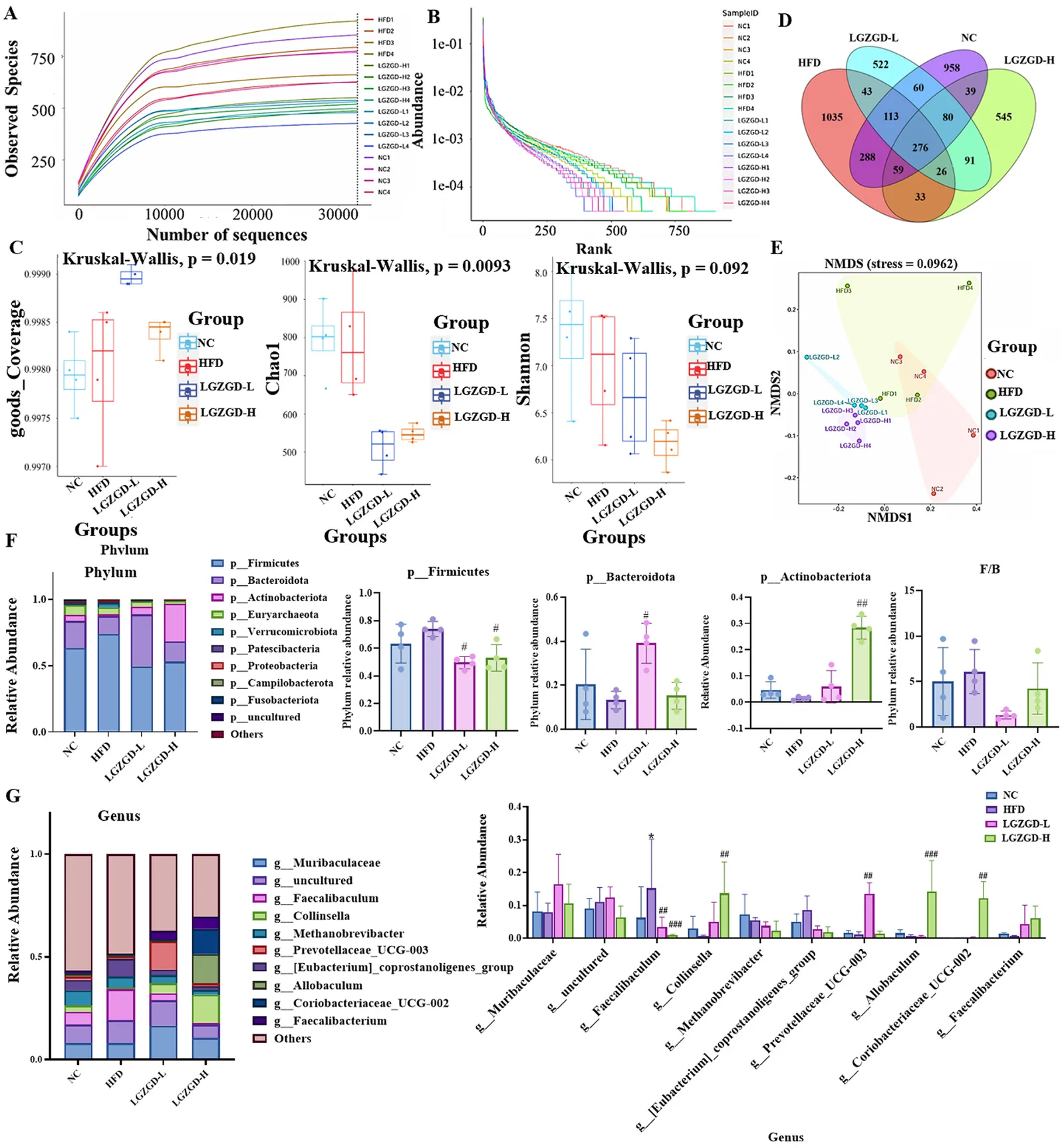
LGZGD improves gut microbiota dysbiosis in MASLD rats. **(A)** Rarefaction curve. **(B)** Rank-Abundance curve. **(C)** Alpha diversity assessed using the Coverage, Chao1, and Shannon indices. **(D)** Venn diagram illustrating the differences in ASV numbers across all groups. **(E)** NMDS analysis. **(F)** Taxonomic composition and dominant microbial communities at the phylum level. **(G)** Taxonomic composition and dominant microbial communities at the genus level (^*^P < 0.05 vs. NC group; ^#^*P* < 0.05, ^*##*^*P* < 0.01, ^*###*^*P* < 0.001 vs. HFD group). ASV, amplicon sequence variant; LGZGD, Ling-Gui-Zhu-Gan Decoction; MASLD, metabolic dysfunction-associated steatotic liver disease; NMDS, non-metric multidimensional scaling.

Fecal samples from each group of rats were subjected to 16S rRNA high-throughput sequencing analysis. The rarefaction and rank-abundance curves indicated that the sequencing depth was sufficient and the data volume was adequate (Figures 3A, B). Based on ASVs, alpha diversity was evaluated using the Coverage, Chao1, and Shannon indices. The results revealed that LGZGD treatment ameliorated the alterations in species diversity and richness induced by HFD (Figure 3C). The Venn diagram illustrates the shared and unique gut microbiota among different groups, revealing that both HFD and LGZGD induced alterations in the gut microbiota (Figure 3D). Non-metric multidimensional scaling (NMDS) analysis showed clear separation among the samples from different groups (Figure 3E). At the phylum level, the dominant bacterial phyla were Firmicutes, Bacteroidetes, and Actinobacteria. Compared with the NC group, the HFD group showed an increased abundance of Firmicutes and decreased abundances of Bacteroidetes and Actinobacteria, resulting in an elevated Firmicutes/Bacteroidetes (F/B) ratio, although these differences were not statistically significant (Figure 3F). Following LGZGD treatment, the abundance of Firmicutes was significantly reduced (*p* < 0.05), while the abundances of Bacteroidetes and Actinobacteria increased (Figure 3F). At the genus level, comparison between the HFD group and the LGZGD-L or LGZGD-H groups identified *g__Faecalibaculum, g__Collinsella*, and *g__Prevotellaceae_UCG-003* as the top three differential dominant genera (Figure 3G). These results indicate that LGZGD ameliorates HFD-induced gut microbiota dysbiosis and restores alterations in microbial composition in MASLD rats.

### 3.4 LGZGD ameliorated HFD-induced disorders of lipid metabolism

To investigate the metabolic differences among different groups, we performed OPLS-DA on the metabolite profiles of serum, liver, and BAT. The results consistently revealed marked differences in metabolic profiles between the NC, HFD, and each drug-treated group, as evidenced by distinct intra-group clustering and clear inter-group separation (Supplementary Figures S2A, S3A, S4A). By comparing the changes in volcano plots between the NC and HFD groups and the LGZGD and HFD groups, the number and trends of lipid alterations among the different groups can be clearly elucidated (Supplementary Figures S2B, S3B, S4B).

#### 3.4.1 Screening of differential lipids in serum

**Figure 4:**
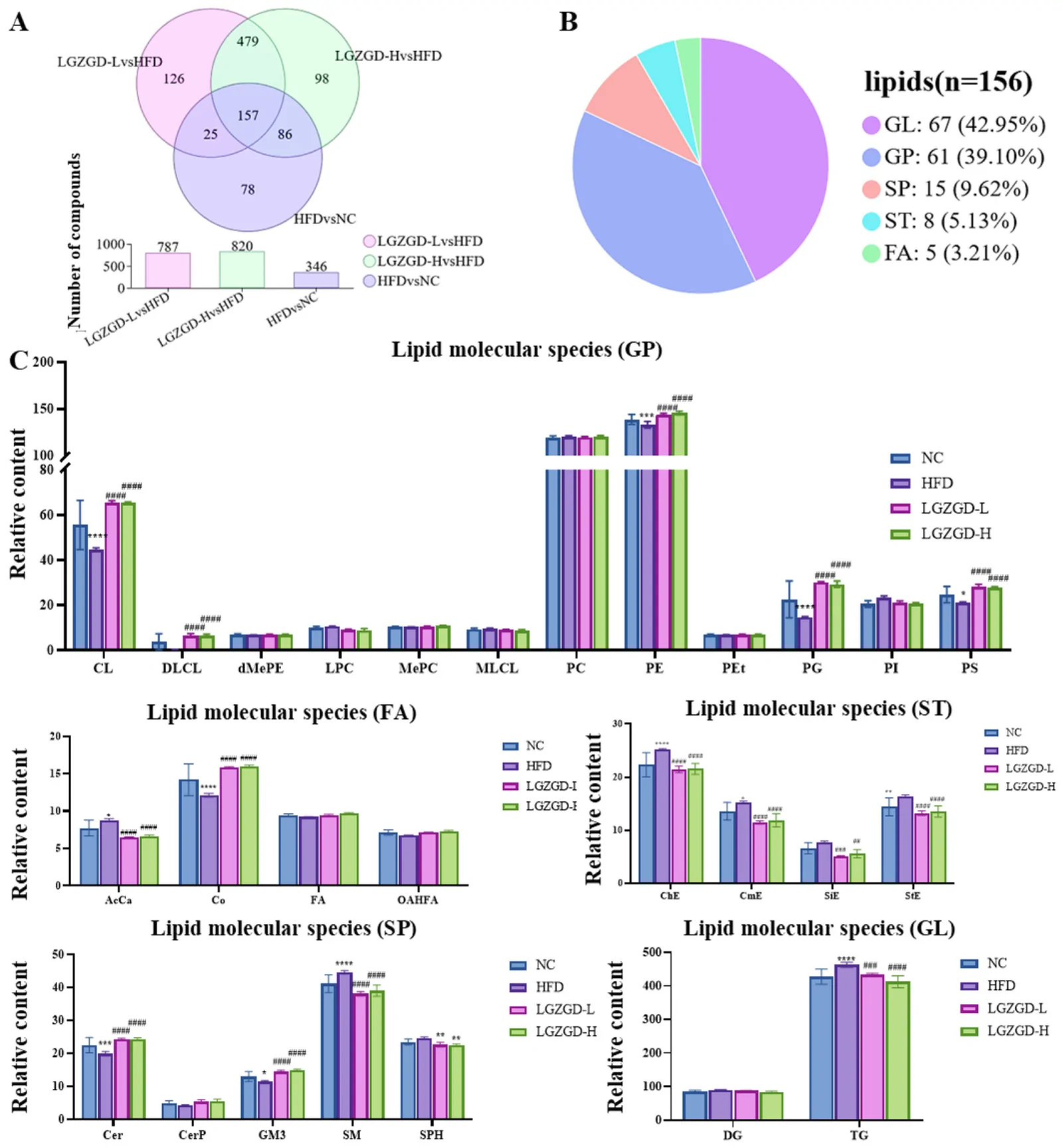
LGZGD ameliorates HFD-induced disruption in serum lipid metabolism. **(A)** Venn diagram of differentially-expressed lipids between HFD vs. NC group and LGZGD vs. HFD group. **(B)** Comparison of lipid classes between HFD vs. NC group and LGZGD vs. HFD group. **(C)** Lipid subclasses analysis. (*n* = 6, *p < 0.05, **p < 0.01, ***p < 0.001, ****p < 0.0001 vs. NC group; ^#^*p* < 0.05; ^##^*p* < 0.01, ^###^*p* < 0.001, ^####^*p* < 0.0001 vs. HFD group). LGZGD, Ling-Gui-Zhu-Gan Decoction; HFD, high-fat diet; NC, normal control. HFD, high-fat diet; LGZGD, Ling-Gui-Zhu-Gan Decoction; NC, normal control.

The intersection of DALs selected from LGZGD-L vs. HFD, LGZGD-H vs. HFD, and HFD vs. NC yielded 157 common DALs (Figure 4A). Among these, 156 displayed opposite trends, primarily including glycerolipids (GL), glycerophospholipids (GP), sphingolipids (SP), sterols (ST), and fatty acyls (FA; Figure 4B), which may serve as metabolic markers for LGZGD in alleviating MASLD. Further analysis of lipid subclasses revealed that, compared to the NC group, the HFD group exhibited significantly increased levels of TG, sphingomyelin (SM), cholesterol ester (ChE), campesterol ester (CmE), sitosterol ester (SiE), stigmasterol ester (StE), and acyl carnitine (AcCa), while the levels of ceramides (Cer), gangliosides (GM3), coenzyme (Co), cardiolipin (CL), dilysocardiolipin (DLCL), phosphatidylethanolamine (PE), phosphatidylglycerol (PG), and phosphatidylserine (PS) were significantly decreased. These changes were markedly attenuated after LGZGD treatment (Figure 4C).

#### 3.4.2 Screening of differential lipids in liver tissue

**Figure 5:**
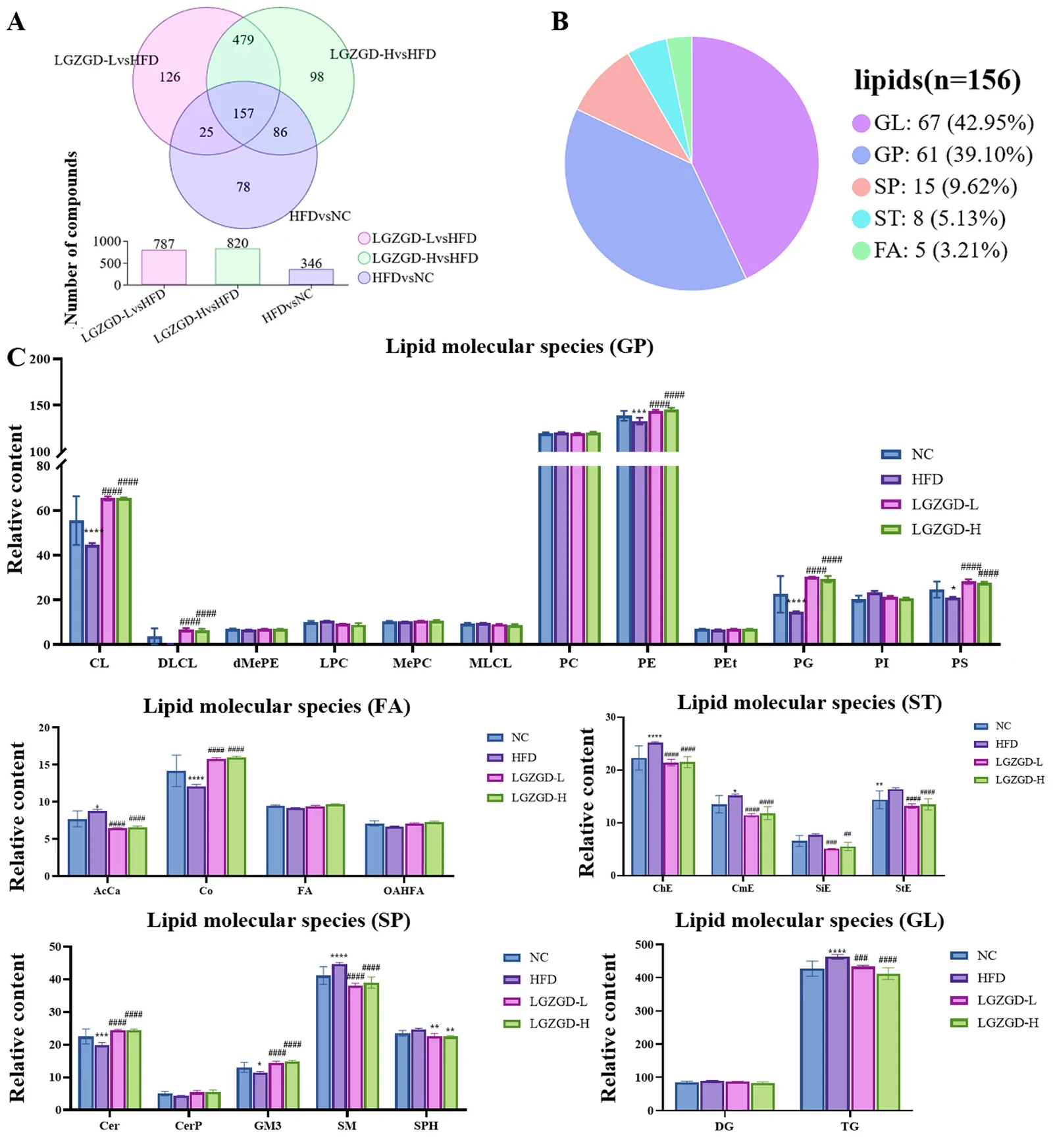
LGZGD ameliorates HFD-induced disruption in hepatic lipid metabolism. **(A)** Venn diagram of differentially-expressed lipids between HFD vs. NC group and LGZGD vs. HFD group. **(B)** Comparison of lipid classes between HFD vs. NC group and LGZGD vs. HFD group. **(C)** Lipid subclasses analysis. (*n* = 6, *p < 0.05, **p < 0.01, ***p < 0.001, ****p < 0.0001 vs. NC group; ^#^*p* < 0.05, ^##^*p* < 0.01, ^###^*p* < 0.001, ^####^*p* < 0.0001 vs. HFD group). HFD, high-fat diet; LGZGD, Ling-Gui-Zhu-Gan Decoction; NC, normal control.

Intersection analysis of DALs from LGZGD-L vs. HFD, LGZGD-H vs. HFD, and HFD vs. NC identified 680 common DALs (Figure 5A), of which 679 exhibited opposite trends (primarily GL, GP, FA, SP, and ST; Figure 5B). These may serve as potential metabolic markers for LGZGD-mediated amelioration of MASLD. Analysis of lipid subclasses showed that a HFD significantly elevated the levels of CL, phosphatidylcholine (PC), PG, Cer, SM, ChE, CmE, StE, zymosterol ester (ZyE), N-acylethanolamine (AEA), OAcyl-(gamma-hydroxy) FA (OAHFA), and wax esters (WE), but reduced the levels of PE, phosphatidylinositol (PI), diglyceride (DG), and TG relative to the NC group. Treatment with LGZGD effectively reversed these HFD-induced alterations (Figure 5C).

#### 3.4.3 Screening of differential lipids in BAT

**Figure 6:**
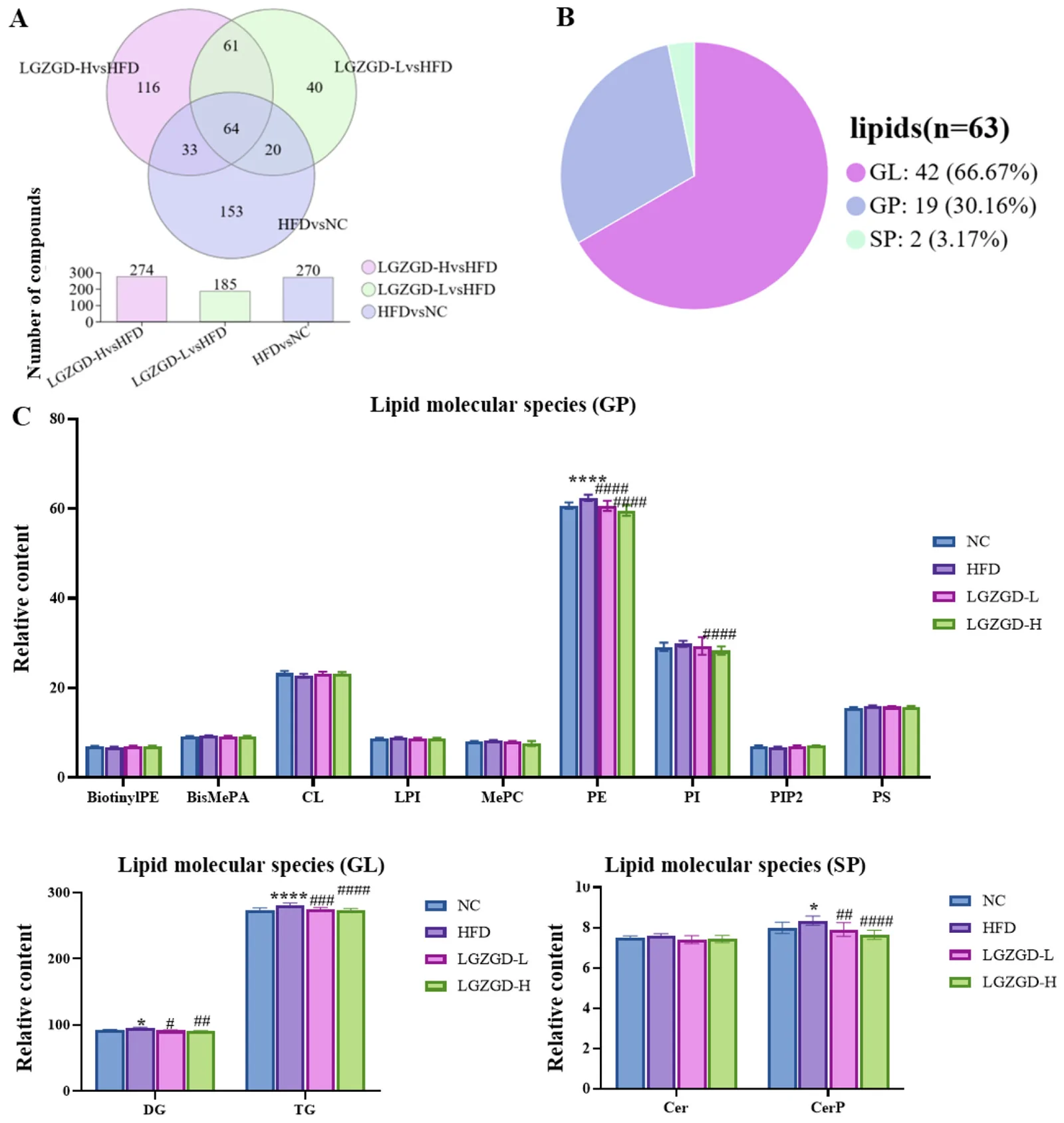
LGZGD ameliorates HFD-induced disruption in BAT lipid metabolism. **(A)** Venn diagram of differentially-expressed lipids between HFD vs. NC group and LGZGD vs. HFD group. **(B)** Comparison of lipid classes between HFD vs. NC group and LGZGD vs. HFD group. **(C)** Lipid subclasses analysis. (*n* = 6, *p < 0.05, ****p < 0.0001 vs. NC group; ^#^*p* < 0.05, ^##^*p* < 0.01, ^###^*p* < 0.001, ^####^*p* < 0.0001 vs. HFD group). HFD, high-fat diet; LGZGD, Ling-Gui-Zhu-Gan Decoction; NC, normal control.

The intersection of DALs from LGZGD-L vs. HFD, LGZGD-H vs. HFD, and HFD vs. NC yielded 64 common DALs (Figure 6A). Among these, 63 displayed opposite trends, primarily including GL, GP, and SP (Figure 6B), which could serve as metabolic markers for LGZGD in alleviating MASLD. Further analysis of lipid subclasses indicated that, relative to the NC group, the HFD group showed significantly elevated relative levels of PE, ceramides phosphate (CerP), DG, and TG. These alterations were notably attenuated following LGZGD administration (Figure 6C).

### 3.5 Metabolic pathway analysis

**Figure 7:**
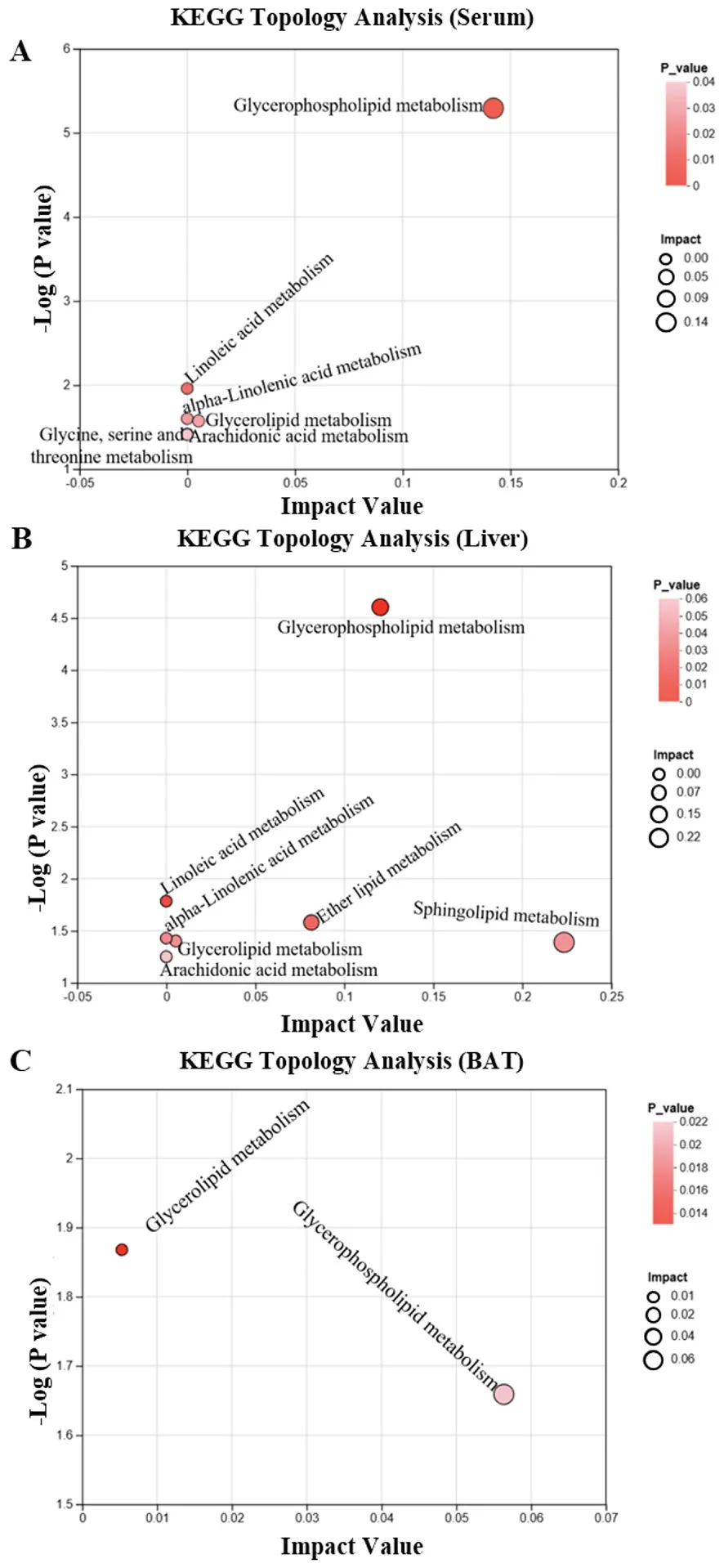
KEGG topology analysis of DALs. **(A)** Serum. **(B)** Liver. **(C)** BAT. In the legend on the right, the size of the black circles indicates the magnitude of Impact: larger circles correspond to larger values. The depth of the red color represents the magnitude of the *p*-value: darker red indicates larger values. DALs, differentially altered lipid metabolites; BAT, brown adipose tissue.

**Figure 8:**
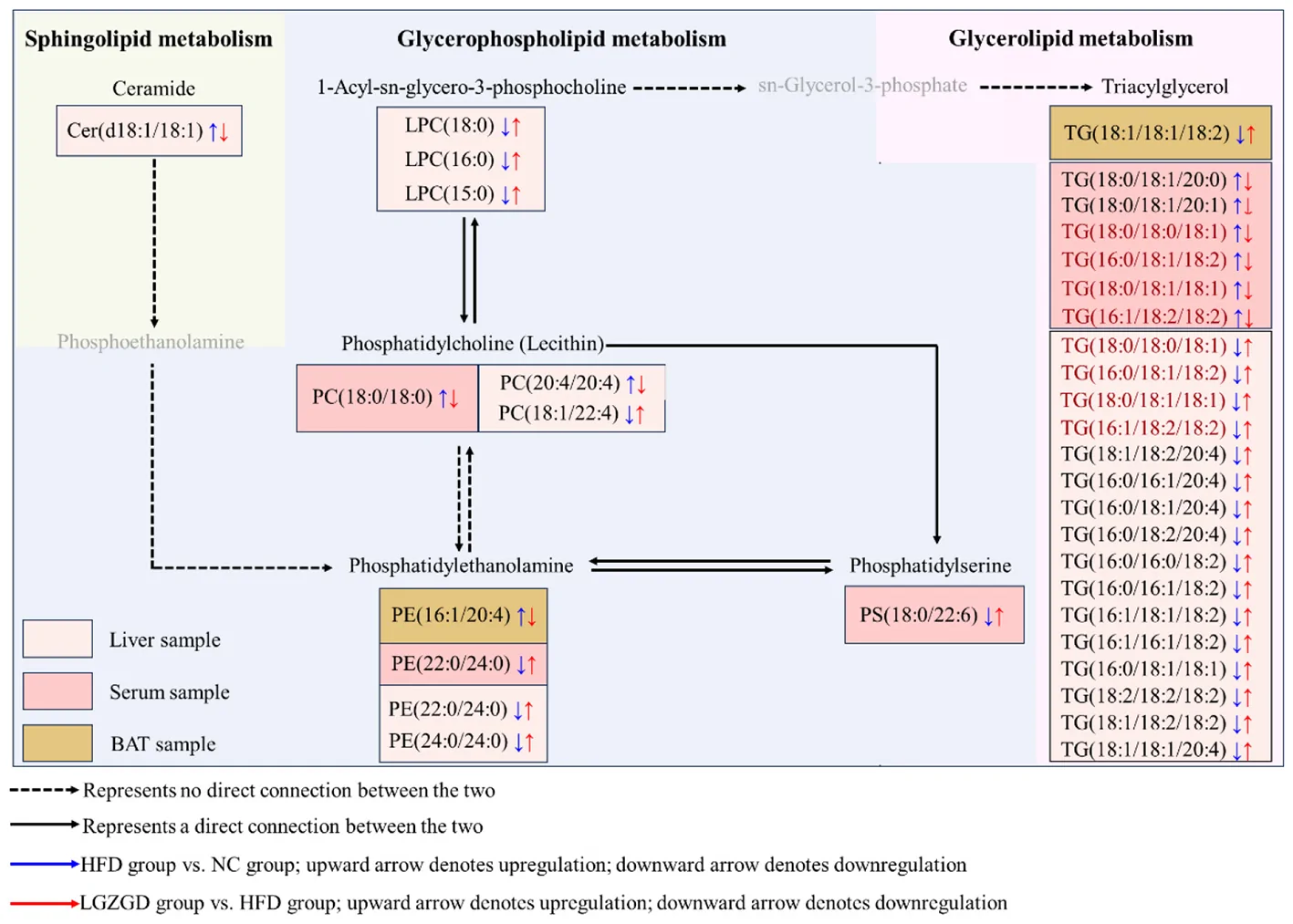
Associations of lipid metabolites across glycerophospholipid, glycerolipid, and sphingolipid metabolism pathways.

KEGG pathway analysis was performed on the DALs identified in serum, liver, and BAT. The results revealed that six metabolic pathways were enriched in serum, seven in the liver, and two in BAT (Figure 7). Glycerophospholipid and glycerolipid metabolism were consistently enriched across all three sample types, characterized by alterations in lipid classes such as PE, PS, PC, LPC, and TG. The sphingolipid metabolism pathway was significantly enriched in liver tissue, with Cer (d18:1/18:1) identified as a key differentially abundant metabolite. Thus, glycerophospholipid, glycerolipid, and sphingolipid metabolisms were identified as the key pathways contributing to the pharmacological effects of LGZGD. These pathways predominantly involved 29 lipid metabolites, including PE(16:1/20:4), PE(22:0/24:0), PE(24:0/24:0), PS(18:0/22:6), PC(18:0/18:0), PC(20:4/20:4), PC(18:1/22:4), LPC(18:0), LPC(16:0), LPC(15:0), TG(18:1/18:1/18:2), TG(18:0/18:1/20:1), TG(18:0/18:1/20:0), TG(18:0/18:0/18:1), TG(16:0/18:1/18:2), TG(18:0/18:1/18:1), TG(16:1/18:2/18:2), TG(18:1/18:2/20:4), TG(16:0/16:1/20:4), TG(16:0/18:1/20:4), TG(16:0/18:2/20:4), TG(16:0/16:0/18:2), TG(16:0/16:1/18:2), TG(16:1/18:1/18:2), TG(16:1/16:1/18:2), TG(16:0/18:1/18:1), TG(18:2/18:2/18:2), TG(18:1/18:2/18:2), TG(18:1/18:1/20:4), and Cer(d18:1/18:1). The specific alterations of these metabolites in serum, liver, and brown adipose tissue samples are presented in Figure 8.

### 3.6 Correlation analysis

To elucidate the potential relationships among multiple variables, we performed an integrative correlation analysis using Cytoscape software (The Cytoscape Consortium, San Diego, CA, USA). This analysis incorporated differential microbiota at the genus level, pharmacodynamic parameters, as well as differentially expressed lipids and associated lipid pathways. The correlation analysis demonstrated that LGZGD alleviated the disease condition in MASLD rats by ameliorating intestinal flora dysbiosis and regulating lipid metabolism levels.

**Figure 9:**
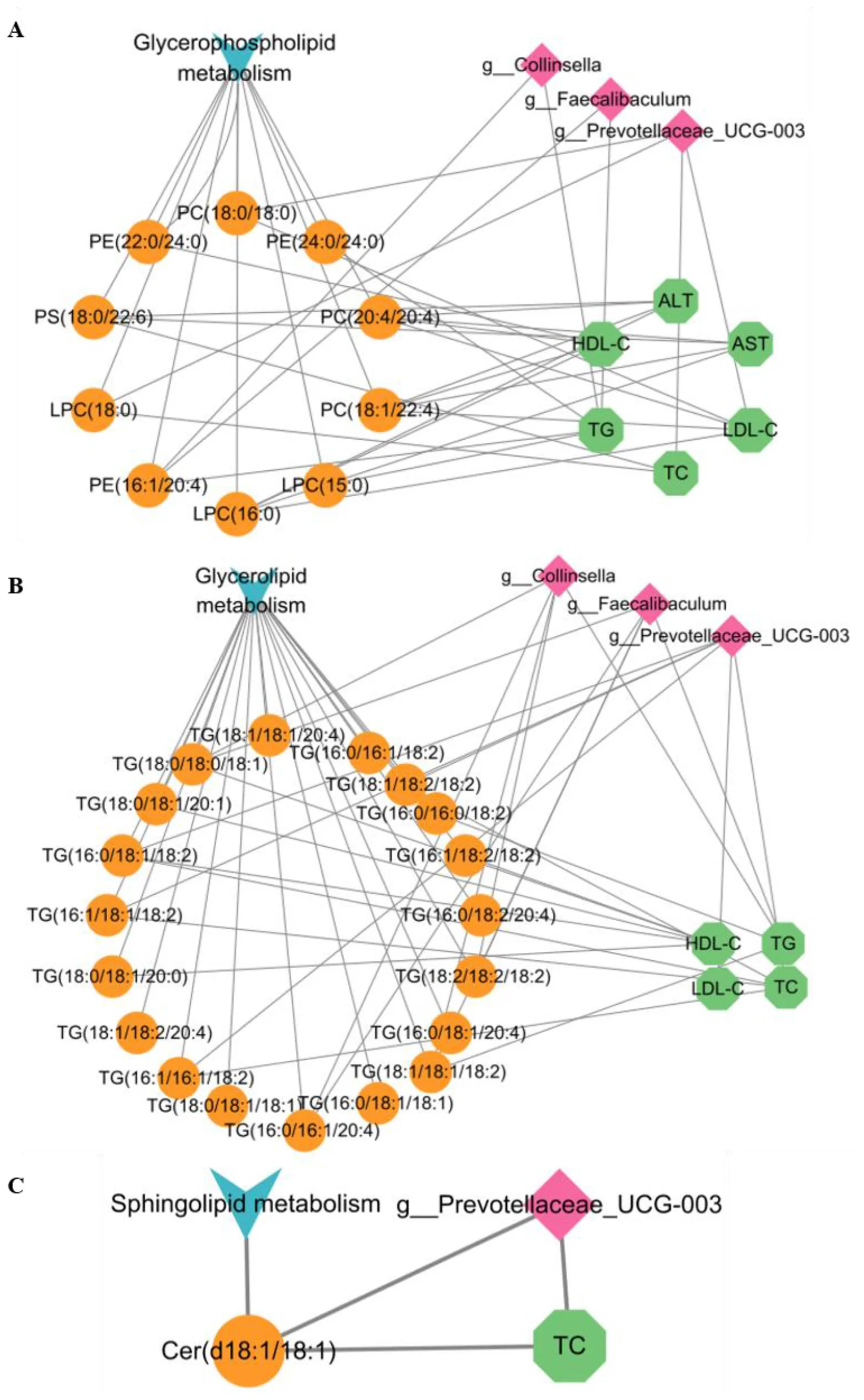
Correlation analysis of differential genus-level bacteria, pharmacodynamic indicators, differential lipids with key metabolic pathway. **(A)** Glycerophospholipid metabolism pathway. **(B)** Glycerolipid metabolism pathway. **(C)** Sphingolipid metabolism pathway.

LGZGD ameliorates MASLD by modulating specific gut microbiota genera, including *g__Prevotellaceae_UCG-003, g__Collinsella*, and *g__Faecalibaculum*, as well as influencing key lipid metabolites within the glycerophospholipid metabolism pathway—such as LPC(18:0), LPC(16:0), LPC(15:s0), PC(18:1/22:4), PC(20:4/20:4), PE(24:0/24:0), PC(18:0/18:0), PS(18:0/22:6), PE(22:0/24:0), and PE(16:1/20:4). These changes subsequently affect relevant physiological parameters including TC, TG, HDL-C, LDL-C (Figure 9A).

LGZGD ameliorates MASLD by modulating specific gut microbiota genera, including *g__Prevotellaceae_UCG-003, g__Collinsella*, and *g__Faecalibaculum*, as well as influencing key lipid metabolites within the glycerolipid metabolism pathway—such as TG(16:0/18:1/18:2), TG(18:1/18:2/20:4), TG(18:0/18:1/18:1), TG(16:0/16:1/20:4), TG(16:0/18:1/20:4), TG(16:0/18:2/20:4), TG(16:0/16:0/18:2), TG(16:0/16:1/18:2), TG(18:0/18:0/18:1), TG(16:1/18:1/18:2), TG(16:1/16:1/18:2), TG(16:0/18:1/18:1), TG(18:2/18:2/18:2), TG(16:1/18:2/18:2), TG(18:1/18:2/18:2), TG(18:1/18:1/20:4), TG(18:0/18:1/20:1), TG(18:0/18:1/20:0), and TG(18:1/18:1/18:2). These changes subsequently affect relevant physiological parameters including TC, TG, HDL-C, LDL-C (Figure 9B).

LGZGD ameliorates MASLD by modulating the gut microbiota genus g__Prevotellaceae_UCG-003 and influencing the lipid metabolite Cer(d18:1/18:1) in the sphingolipid metabolism pathway, thereby regulating the TC levels (Figure 9C).

## 4 Discussion

In this study, a total of 83 compounds were putatively identified in LGZGD using a UPLC-Q-TOF-MS system, including seven derived from PR, three from CR, eight from AM, and 60 from GR, with five compounds shared by two or more herbal components. The main compounds include flavones, terpenoids, organic acids, and phenylpropanoids. Previous studies have reported that the compounds, such as pachymic acid, polyporenic acid C (He et al., 2022; Ren et al., 2025), atractylenolide I (Liu, 2017), liquiritin (Wei, 2023), glycyrrhizic acid (Bailly and Vergoten, 2020; Li et al., 2019), formononetin (Liao et al., 2024), glycycoumarin (Zhang et al., 2020), glycyrrhetinic acid (Fan et al., 2022), and betulinic acid (Zhao et al., 2022) exert ameliorative effects on MASLD. These findings suggest that these compounds may contribute to the potential efficacy of LGZGD against MASLD; however, further bioavailability assessments and *in vitro* activity screening are required to confirm their roles as key active constituents.

MASLD is one of the most prevalent chronic liver diseases worldwide, affecting up to 25% of the global population. In this study, we established HFD-induced MASLD rat model and systematically evaluated the ameliorative effects of LGZGD on MASLD. H&E staining reveals that LGZGD ameliorated hepatic steatosis. Biochemical analysis revealed that, compared with the NC group, the HFD group exhibited elevated serum and hepatic levels of TG, TC, AST, and LDL-C, along with a significant reduction in HDL-C levels. Administration of LGZGD led to decreased levels of TG, TC, ALT, AST, and LDL-C, while significantly increasing HDL-C levels. These findings confirm the successful establishment of the MASLD model and indicate markedly disrupted lipid metabolism in both serum and liver tissues of MASLD rats induced by the HFD. Intervention with LGZGD effectively ameliorated these abnormal lipid profiles.

The gut microbiota plays a crucial role in maintaining host immune and metabolic homeostasis, as well as pathogen resistance. The rarefaction and rank-abundance curves demonstrate sufficient sequencing depth and appropriate sequencing volume. Alpha diversity analysis reveals that LGZGD ameliorated the abnormal species diversity and richness induced by HFD. Beta diversity analysis demonstrates significant intergroup differences in gut microbiota composition, indicating that LGZGD intervention significantly altered the gut microbiota of HFD-induced MASLD model rats. Specifically, it increased the relative abundances of *Collinsella* and *Prevotellaceae_UCG-003* at the genus level, while decreasing the levels of *Faecalibaculum*. Previous studies have demonstrated that *g_Faecalibaculum* modulates intestinal integrity in diabetic mice by regulating the expression of tight junction proteins (Min et al., 2014). *g__Collinsella* has also been reported to produce short-chain fatty acids (SCFAs) (Yin et al., 2013). *g__Prevotellaceae_UCG-003*, which is associated with short-chain fatty acid production, has the potential to regulate intestinal inflammation (Gao et al., 2022). Following LGZGD administration, observations at the genus level were consistent with these earlier findings, further confirming the important role of these microbial communities in maintaining gut homeostasis and influencing host metabolic health. These findings collectively indicate that LGZGD ameliorates MASLD by improving gut microbiota dysbiosis.

Lipids are essential metabolites in living organisms, performing diverse biological functions and participating in critical physiological processes such as cellular energy storage, membrane structure formation, and signal transduction. To further elucidate the underlying metabolic mechanisms, we employed a lipidomics approach to investigate the ameliorative effects of LGZGD in MASLD rats. Serum, liver, and interscapular BAT lipidomics analysis identified 156, 679, and 63 DALs, respectively. After removing duplicates, LGZGD was found to primarily regulate 831 lipids, which were predominantly classified into the following categories: GL, GP, SP, ST, and FA. Following LGZGD treatment, the altered lipid profiles showed a trend of reversal toward the NC group levels, suggesting that LGZGD may ameliorate MASLD through the modulation of lipid metabolism. KEGG pathway enrichment analysis of DALs revealed that glycerophospholipid, glycerolipid, and sphingolipid metabolism were the key pathways involved in the pharmacological effects of LGZGD. These pathways primarily involved 29 lipid metabolites, including PE(16:1/20:4), PE(22:0/24:0), PE(24:0/24:0), PS(18:0/22:6), PC(18:0/18:0), PC(20:4/20:4), PC(18:1/22:4), LPC(18:0), LPC(16:0), LPC(15:0), TG(18:1/18:1/18:2), TG(18:0/18:1/20:1), TG(18:0/18:1/20:0), TG(18:0/18:0/18:1), TG(16:0/18:1/18:2), TG(18:0/18:1/18:1), TG(16:1/18:2/18:2), TG(18:1/18:2/20:4), TG(16:0/16:1/20:4), TG(16:0/18:1/20:4), TG(16:0/18:2/20:4), TG(16:0/16:0/18:2), TG(16:0/16:1/18:2), TG(16:1/18:1/18:2), TG(16:1/16:1/18:2), TG(16:0/18:1/18:1), TG(18:2/18:2/18:2), TG(18:1/18:2/18:2), TG(18:1/18:1/20:4), and Cer(d18:1/18:1). These metabolites encompass both saturated and unsaturated fatty acids with heterogeneous chain lengths, and play pivotal regulatory roles in inflammatory responses and lipid metabolism. PC, PE, and PS are all components of glycerophospholipids and are associated with a variety of metabolic diseases. PE has been demonstrated to regulate cellular metabolism, oxidative stress, and inflammatory signaling in both cellular and animal models (Petkevicius et al., 2022). PC has also been shown to be closely associated with the development of MASLD (Wang et al., 2021). PS participates in the signaling regulation of growth and development and can be enzymatically converted into PE, thereby providing resources for the synthesis of other lipids (Kim et al., 2014). LPC (16:0) has been reported to restore glucose uptake in insulin-resistant adipocytes (Goto, 2019), while LPC (18:0) has also exhibited anti-MASLD effects (Luo et al., 2018). TG, which is considered to be associated with numerous diseases such as cardiovascular disease, ischemic stroke, and dyslipidemia, is an important glyceride involved in the process of MAFLD (Sun et al., 2020). Cer is a central molecule in sphingolipid metabolism and is closely associated with inflammatory responses. These findings suggest that LGZGD ameliorates MASLD by modulating lipid metabolism.

The correlation analysis revealed that gut microbial genera such as Prevotellaceae_UCG-003, Collinsella, and Faecalibaculum were strongly correlated with multiple lipid metabolic pathways, including glycerophospholipid metabolism, glycerolipid metabolism, and sphingolipid metabolism, as well as with pharmacodynamic indices. Therefore, we hypothesize that LGZGD may exert its preventive effects against MASLD through coordinated regulation of gut microbiota composition and the associated lipid metabolic network.

Admittedly, several limitations of the present study should be acknowledged and addressed in future work. For instance, although we employed both low and high dosages of LGZGD in the animal experiments, toxicological evaluations of these doses were not performed. Moreover, while our findings confirm that LGZGD improves lipid parameters in MASLD rats through modulation of gut microbiota and lipid metabolism–related pathways, the causal relationships among these effects remain to be established. Therefore, additional experiments—such as toxicological assessments, fecal microbiota transplantation, or targeted metabolite intervention—are warranted in subsequent studies to clarify the safety profile and underlying mechanisms of LGZGD. Furthermore, the sample size in this study was relatively small, and all results should be considered exploratory; they await validation in larger cohort studies.

## 5 Conclusion

LGZGD exerts a hepatoprotective effect in MASLD rats. The underlying mechanism may involve the modulation of gut microbiota, including the genera *Prevotellaceae_UCG-003, Faecalibaculum, and Collinsella*; and the regulation of lipid metabolism pathways such as glycerophospholipid, glycerolipid, and sphingolipid metabolism.

## Data Availability

The raw 16S rRNA sequences are available in the NCBI database under the BioProject number PRJNA1454296, with biosamples number SAMN57297327 to SAMN57297342.
