# Supplementary material for: Unraveling the ameliorative effects of Ling-Gui-Zhu-Gan decoction on MASLD: an integrative gut microbiota and lipidomic study
Source: Front Microbiol. 2026 Sep 3;17:1916850. doi: 10.3389/fmicb.2026.1916850 (PMC13582187; doi:10.3389/fmicb.2026.1916850)
Supplement: Supplementary file 1 [file Supplementary_File_1.docx]

**Electronic Supplementary Material**

**Unraveling the Therapeutic Action of Ling-Gui-Zhu-Gan Decoction on MASLD: An Integrative Gut Microbiota and Lipidomic Study**

Juanhong Lei ^a, c,^ ^1^, Dihong Gong ^b, c, 1^, Yaqin Yang ^b, c^, Li Li ^a, c^, Yue Zhou ^a, c^, Guanrong Qiao ^b, c^, Xingxin Yang ^b, c, *^, Jie Yu ^b, c, *^

^a^ The First Clinical College, Yunnan University of Chinese Medicine, Kunming 650500, China;

^b^ College of Pharmaceutical Science, Yunnan University of Chinese Medicine, Kunming 650500, China;

^c^ Yunnan Key Laboratory of Southern Medicine Utilization, Kunming 650500, China;

^1^ These authors contributed equally to this work.

^*^ **Corresponding authors**

E-mail addresses: yxx78945@163.com (X. Yang), yujie@ynucm.edu.cn (J. Yu).

**Table of Contents**

**Supplementary Figures and Table**

**1. Figure S1.** Base peak chromatograms of LGZGD extract. (A) Positive ionization modes. (B) Negative ionization modes.

**2. Figure S2.** LGZGD effectively ameliorated the disruption of serum lipid metabolism induced by HFD. (A) OPLS-DA score plot and permutation test plot (after 200 times). (B) Volcano plot of differential lipid metabolites.

**3. Figure S3.** LGZGD effectively ameliorated the disruption of hepatic lipid metabolism induced by HFD. (A) OPLS-DA score plot and permutation test plot (after 200 times). (B) Volcano plot of differential lipid metabolites.

**4. Figure S4.** LGZGD effectively ameliorated the disruption of BAT lipid metabolism induced by HFD. (A) OPLS-DA score plot and permutation test plot (after 200 times). (B) Volcano plot of differential lipid metabolites.

**5. Table S1.** Composition of LGZGD

**6. Table S2.** Identification of chemical constituents in LGZGD.

**Supplementary Figures and Table**

A

B

**Figure S1.** Base peak chromatograms of LGZGD extract. (A) Positive ionization modes. (B) Negative ionization modes.

A

B

**Figure S2.** LGZGD effectively ameliorated the disruption of serum lipid metabolism induced by HFD. (A) OPLS-DA score plot and permutation test plot (after 200 times). (B) Volcano plot of differential lipid metabolites. HFD, high-fat diet; LGZGD, Ling-Gui-Zhu-Gan Decoction; OPLS-DA, Orthogonal partial least squares-discriminant analysis.

A

B

**Figure S3.** LGZGD effectively ameliorated the disruption of hepatic lipid metabolism induced by HFD. (A) OPLS-DA score plot and permutation test plot (after 200 times). (B) Volcano plot of differential lipid metabolites. HFD, high-fat diet; LGZGD, Ling-Gui-Zhu-Gan Decoction; OPLS-DA, Orthogonal partial least squares-discriminant analysis.

A

B

**Figure S4.** LGZGD effectively ameliorated the disruption of BAT lipid metabolism induced by HFD. (A) OPLS-DA score plot and permutation test plot (after 200 times). (B) Volcano plot of differential lipid metabolites. HFD, high-fat diet; LGZGD, Ling-Gui-Zhu-Gan Decoction; OPLS-DA, Orthogonal partial least squares-discriminant analysis.

**Table S1.** Composition of LGZGD

| **No.** | **Medicinal Latin Name** | **Botanical Latin Name** | **Chinese Pinyin Name** | **Abbreviation** |
| --- | --- | --- | --- | --- |
| 1 | *Poria* | *Poria* *cocos*（Schw.）Wolf | Fuling | PR |
| 2 | *Cinnamomi Ramulus* | *Cinnamomum cassia* Presl | Guizhi | CR |
| 3 | *Atractylodis Macrocephalae Rhizoma* | *Cinnamomum cassia* Presl | Baizhu | AM |
| 4 | *Glycyrrhizae Radix et Rhizoma* | *Glycyrrhiza uralensis* Fisch., *Glycyrrhiza inflata* Bat. and *Glycyrrhiza glabra* L. | Gancao | GR |

**Table S2.** Identification of chemical constituents in LGZGD

| **NO.** | **Ion Mode** | **tR /min** | **Molecular formula** | **Theoretical molecular weight m/z** | **Actual molecular weight m/z** | **δ/ppm** | **Fragment ions (m/z)** | **Compound name** | **Source** | **Type** |
| --- | --- | --- | --- | --- | --- | --- | --- | --- | --- | --- |
| 1 | ESI^-^ | 1.418 | C_7_H_12_O_6_ | 191.0561 | 191.0564 | -1.51 | 172.9838、126.9823、110.9891、92.9808、84.9770、128.9611、86.9561 | D-(-)-Quinic acid | PR | OA |
| 2 | ESI^+^ | 1.482 | C_12_H_22_O_11_ | 365.1054 | 365.1056 | -0.46 | 203.0528、185.0413 | Sucrose/ Maltose | PR、CR、AM、GR | O |
|  | ESI^-^ | 1.276 |  | 341.1089 | 341.1091 | -0.48 | 119.0052、179.0246、149.0145、131.0046 |  |  |  |
| 3 | ESI^+^ | 1.958 | C_7_H_6_O_2_ | 123.0441 | 123.044 | 0.45 | / | Benzoic acid | AM | OA |
| 4 | ESI^+^ | 1.998 | C_5_H_5_N_5_O | 152.056 7 | 152.0567 | -0.09 | 110.9621、135.0163 | Guanine | GR | O |
| 5 | ESI^+^ | 2.162 | C_6_H_13_NO_2_ | 132.1019 | 132.102 | -0.72 | 132.102 | l-isoleucine | PR、AM | O |
| 6 | ESI^+^ | 2.238 | C_8_H_9_N | 120.0808 | 120.0808 | -0.2 | 103.0267、91.0276 | N-Benzylidenemethylamine | GR | O |
| 7 | ESI^+^ | 2.258 | C_5_H_5_N_5_ | 136.061 8 | 136.0618 | -0.21 | 119.0349 | Adenine | PR、GR | O |
| 8 | ESI^-^ | 3.249 | C_13_H_16_O_9_ | 315.0722 | 315.0724 | -0.78 | 108.0009 | Protocatechuic acid glucoside | GR、AM、CR | G |
| 9 | ESI^-^ | 4.849 | C_22_H_38_O_12_ | 493.2291 | 493.2295 | -0.91 | 447.3321、143.0350、119.0341、89.0235、71.0132、59.0135 | (IR,4S,6R}-1,3,3-trimethyl-2-oxabicyclo [2.2.2] oct-6-yl-6-O-B-D-glucopyranosyl-B-D-glucopyranoside | AM | G |
| 10 | ESI^-^ | 5.541 | C_9_H_10_O_3_ | 165.0557 | 165.0559 | -1.1 | 119.0603、59.0219、93.0442、121.0763 | Desaminotyrosine | GR | OA |
| 11 | ESI^+^ | 5.637 | C_7_H_6_O | 107.0497 | 107.049 | 1.32 | 79.0541、77.0384 | Benzaldehyde | CR | O |
| 12 | ESI^+^ | 7.032 | C_6_H_6_O_3_ | 127.039 | 127.0389 | 0.56 | 109.0284 | 5-Hydroxymethylfurfural | AM | O |
| 13 | ESI^+^ | 8.04 | C_18_H_16_O_4_ | 297.1121 | 297.112 | 0.46 | 279.1013、235.0765 | Truxinic acid or isomer | CR | OA |
| 14 | ESI^-^ | 8.473 | C_9_H_10_O_3_ | 165.0557 | 165.0559 | -1.1 | 121.0661 | Phenyllactic acid | GR | OA |
| 15 | ESI^-^ | 9.159 | C_26_H_28_O_14_ | 563.1406 | 563.1407 | -0.13 | 503.1046、473.0947、443.0869、383.0635、353.0525 | Schaftoside | GR | F |
| 16 | ESI^+^ | 10.289 | C_11_H_12_O_3_ | 193.0495 | 193.0495 | 0.18 | 178.0260、133.0288 | (E)-Ethyl 3-(4-hydroxyphenyl) acrylate | AM | O |
| 17 | ESI^+^ | 10.393 | C_15_H_12_O_5_ | 273.0757 | 273.0757 | 0.18 | 153.0179、119.0484 | Naringenin | GR | F |
| 18 | ESI^-^ | 10.911 | C_26_H_30_O_13_ | 549.1614 | 549.1614 | -0.06 | 417.0934、255.0243、119.0045 | Liquiritin apioside | GR | F |
| 19 | ESI^-^ | 10.436 | C_21_H_22_O_10_ | 433.114 | 433.1139 | 0.28 | 271.0611、151.0033 | Choerospondin/Prunin /Naringenin-7-O-glucoside | GR | F |
| 20 | ESI^+^ | 10.506 | C_27_H_30_O_14_ | 579.1708 | 579.1702 | 1.09 | 379.0813 | Violanthin | GR | F |
|  | ESI^-^ | 10.554 |  | 577.1563 | 577.1562 | 0.14 | 457.1011、383.0640、353.0530、325.0584 |  |  |  |
| 21 | ESI^+^ | 10.84 | C_15_H_12_O_4_ | 257.0808 | 257.0807 | 0.53 | 137.0233、119.0492 | Liquiritigenin/ Isoliquiritigenin | GR | F |
| 22 | ESI^-^ | 11.104 | C_21_H_22_O_9_ | 417.119 1 | 417.119 | 0.25 | 255.0316、119.0164 | Liquiritin | GR | F |
| 23 | ESI^-^ | 13.359 | C_26_H_30_O_14_ | 565.156 3 | 565.156 | 0.49 | 271.0271 | Cassiaside B | GR | F |
| 24 | ESI^+^ | 13.946 | C_9_H_6_O_2_ | 147.0441 | 147.0439 | 1.06 | 103.0540、91.0543 | Coumarin | PR、CR、AM | P |
| 25 | ESI^+^ | 15.079 | C_15_H_12_O_4_ | 257.0808 | 257.0807 | 0.53 | 137.0234、119.0487 | Liquiritigenin/ Isoliquiritigenin | GR | F |
| 26 | ESI^-^ | 15.367 | C_26_H_30_O_13_ | 549.1614 | 549.1614 | -0.06 | 255.0664、429.1183 | Isoliquiritin apioside/ Liguiritigenin-7-O-D-apiosyl-4'-O-D-glucoside or isomer | GR | F |
| 27 | ESI^-^ | 15.65 | C_27_H_30_O_13_ | 561.1614 | 561.161 | 0.65 | 267.0658、252.0423 | Glycyroside | GR | F |
| 28 | ESI^+^ | 15.839 | C_15_H_12_O_4_ | 257.0808 | 257.0807 | 0.53 | 137.0233、119.0492 | Liquiritigenin/ Isoliquiritigenin | GR | F |
| 29 | ESI^-^ | 16.143 | C_26_H_30_O_13_ | 549.1614 | 549.1614 | -0.06 | 255.0664、429.1046 | Isoliquiritin apioside/ Liguiritigenin-7-O-D-apiosyl-4'-O-D-glucoside or isomer | GR | F |
| 30 | ESI^+^ | 16.144 | C_16_H_12_O_4_ | 269.0808 | 269.0806 | 0.87 | 254.0574、226.0625、197.0597、118.0413、237.0543、213.0912 | Isoformononetin/ Formononetin | GR | F |
| 31 | ESI^-^ | 16.149 | C_21_H_22_O_9_ | 417.119 1 | 417.119 | 0.25 | 255.0672、135.0090、119.0501、91.0190、148.0167 | Neoliquiritin/ Neoisoliquiritin/ Isoliquiritoside/ or isomer | GR | F |
| 32 | ESI^+^ | 16.156 | C_22_H_22_O_9_ | 431.1337 | 431.1331 | 1.3 | 269.0809 | Ononin | GR | F |
| 33 | ESI^-^ | 16.426 | C_16_H_12_O_4_ | 267.0663 | 267.066 | 1.06 | 252.0427、132.0217 | Pallidiflorin | GR | F |
| 34 | ESI^-^ | 17.016 | C_21_H_22_O_9_ | 417.119 1 | 417.119 | 0.25 | 255.0663、135.0090、119.0502、91.0195、148.0165 | Neoliquiritin/ Neoisoliquiritin/ Isoliquiritoside/ or isomer | GR | F |
| 35 | ESI^+^ | 17.216 | C_15_H_12_O_4_ | 257.0808 | 257.0807 | 0.53 | 137.0174、119.0430 | Liquiritigenin/ Isoliquiritigenin | GR | F |
| 36 | ESI^-^ | 17.385 | C_35_H_36_O_15_ | 695.1981 | 695.1972 | 1.36 | 549.1625、399.1064、255.0662 | Uralenoside B/ Licoraside D2 / D1 | GR | F |
| 37 | ESI^+^ | 28.872 | C_15_H_12_O_4_ | 257.0808 | 257.0807 | 0.53 | 137.0233、119.0492 | Liquiritigenin/ Isoliquiritigenin | GR | F |
| 38 | ESI^+^ | 18.639 | C_16_H_12_O_5_ | 285.0757 | 285.0757 | 0.18 | 269.0441、270.0523、253.0498、137.0233、257.0818 | Calycosin | GR | F |
| 39 | ESI^-^ | 22.317 | C_42_H_62_O_17_ | 837.3914 | 837.3908 | 0.75 | 351.0569 | Licoricesaponin P2/ Licoricesaponin G2/ 24-Hydroxyglycyrrhizic acid/ Licoricesaponin Q2/ Uralsaponin N/ Yunganoside K2 or isomer | GR | T |
| 40 | ESI^+^ | 22.947 | C_10_H_8_O_2_ | 161.0597 | 161.0597 | 0.04 | 161.0597 | 6-Methylcoumarin | CR | P |
| 41 | ESI^+^ | 23.371 | C_16_H_14_O_4_ | 271.0965 | 271.0962 | 1.05 | 229.1103、177.0786、121.0509、107.0710 | 3-Deoxysappanchalcone | GR | F |
| 42 | ESI^-^ | 25.106 | C_42_H_62_O_18_ | 853.3863 | 853.3854 | 1.1 | 351.0569 | 22 hydroxy licoricesaponin G2 | GR | T |
| 43 | ESI^+^ | 26.785 | C_48_H_72_O_21_ | 985.463 9 | 985.4616 | 2.32 | 809.4305、633.3981、615.3899、453.3371、471.3458、647.3792 | Licoricesaponin A3 | GR | T |
|  | ESI^-^ | 26.931 |  | 983.449 3 | 983.4485 | 0.85 | 821.3961、351.0568、823.3992 |  |  |  |
| 44 | ESI^+^ | 26.791 | C_20_H_36_O_7_ | 389.2534 | 389.2541 | -1.85 | 371.2443、330.2066、284.2006、256.2054、244.1712、232.1701 | 8,9-Epoxy atracolactone | AM | S |
| 45 | ESI^-^ | 28.236 | C_44_H_64_O_18_ | 879.402 | 879.401 | 1.12 | 351.0558、193.0353 | 22-Beta-Acetoxyglycyrrhizin | GR | T |
| 46 | ESI^-^ | 29.482 | C_42_H_62_O_17_ | 837.3914 | 837.3908 | 0.75 | 351.0569 | Licoricesaponin P2/ Licoricesaponin G2/ 24-Hydroxyglycyrrhizic acid / Licoricesaponin Q2/ Uralsaponin N/ Yunganoside K2 or isomer | GR | T |
| 47 | ESI^+^ | 29.552 | C_16_H_12_O_4_ | 269.0808 | 269.0806 | 0.87 | 254.0572、226.0618、197.0602、118.0416、237.0545、213.0907 | Isoformononetin/ Formononetin | GR | F |
| 48 | ESI^+^ | 33.248 | C_42_H_64_O_16_ | 825.4267 | 825.4248 | 2.32 | 455.3434 | Uralsaponin P | GR | T |
| 49 | ESI^-^ | 33.298 | C_42_H_62_O_17_ | 837.3914 | 837.3908 | 0.75 | 351.0496 | Licoricesaponin P2/ Licoricesaponin G2/ 24-Hydroxyglycyrrhizic acid / Licoricesaponin Q2/ Uralsaponin N/ Yunganoside K2 or isomer | GR | T |
| 50 | ESI^-^ | 35.203 | C_42_H_62_O_17_ | 837.3914 | 837.3908 | 0.75 | 351.203 | Licoricesaponin P2/ Licoricesaponin G2/ 24-Hydroxyglycyrrhizic acid / Licoricesaponin Q2/ Uralsaponin N/ Yunganoside K2 or isomer | GR | T |
| 51 | ESI^+^ | 37.245 | C_21_H_20_O_6_ | 369.133 3 | 369.1325 | 2.07 | 285.0771、313.0706 | Gancaonin B/N / Glicoricone | GR | F |
| 52 | ESI^-^ | 38.123 | C_42_H_62_O_17_ | 837.3914 | 837.3908 | 0.75 | 351.0564 | Licoricesaponin P2/ Licoricesaponin G2/ 24-Hydroxyglycyrrhizic acid / Licoricesaponin Q2/ Uralsaponin N or isomer | GR | T |
| 53 | ESI^+^ | 38.664 | C_30_H_44_O_3_ | 453.3363 | 453.3361 | 0.49 | 407.3307、435.3257 | Melliferone or isomer | GR | O |
| 54 | ESI^+^ | 38.777 | C_42_H_62_O_16_ | 823.4111 | 823.4095 | 1.9 | 647.3787、471.3465、453.3365 | Glycyrrhizic acid* | GR | T |
|  | ESI^-^ | 38.701 |  | 821.396 5 | 821.396 | 0.62 | 645.3649、469.3337、803.3838、759.3985、351.0566 |  |  |  |
| 55 | ESI^+^ | 40.839 | C_30_H_44_O_3_ | 453.3363 | 453.3361 | 0.49 | 407.3299、435.3296 | Melliferone or isomer | GR | O |
| 56 | ESI^-^ | 44.149 | C_42_H_64_O_15_ | 807.4172 | 807.4165 | 0.92 | 351.0578 | Licoricesaponin B2 or isomer | GR | T |
| 57 | ESI^+^ | 44.241 | C_15_H_18_O_2_ | 231.138 | 231.1376 | 1.54 | 185.1324、143.0850、117.0699、105.0689、91.0543 | Atractylenolide I | AM | S |
| 58 | ESI^-^ | 46.253 | C_42_H_62_O_16_ | 821.396 5 | 821.396 | 0.62 | 351.0565 | Licoricesaponin K2/ Uralsaponin B/ Licoricesaponin H2/ Licoricesaponin K3/ Uralsaponin A/ Dipotassium glycyrrhizinate/ Diammonium glycyrrhizinate or isomer | GR | T |
| 59 | ESI^-^ | 46.967 | C_20_H_18_O_6_ | 353.1031 | 353.1026 | 1.31 | 175.0401 | Gancaonin L/ Licoisoflavanone | GR | F |
| 60 | ESI^-^ | 48.015 | C_42_H_62_O_16_ | 821.396 5 | 821.396 | 0.62 | 113.0245、193.0353、351.0565、759.3957 | Licoricesaponin K2/ Uralsaponin B/ Licoricesaponin H2/ Licoricesaponin K3/ Uralsaponin A/ Dipotassium glycyrrhizinate/ Diammonium glycyrrhizinate or isomer | GR | T |
| 61 | ESI^+^ | 48.737 | C_21_H_20_O_6_ | 369.133 3 | 369.1325 | 2.07 | 285.0748、313.0707、270.0549 | Glycycoumarin | GR | P |
|  | ESI^-^ | 48.98 |  | 367.118 7 | 367.1183 | 1.12 | 227.0360、309.0404、297.0399、284.0332、203.0715 |  |  |  |
| 62 | ESI^+^ | 50.437 | C_21_H_24_O_5_ | 357.1697 | 357.1688 | 2.38 | 301.1076、123.0441、221.1169 | Glyasperin C | GR | F |
|  | ESI^-^ | 50.68 |  | 355.1551 | 355.1547 | 1.12 | 245.1183、323.1292、283.0615、203.0708、135.0452 |  |  |  |
| 63 | ESI^-^ | 50.533 | C_20_H_18_O_6_ | 353.1031 | 353.1026 | 1.31 | 175.0409 | Gancaonin L/ Licoisoflavanone | GR | F |
| 64 | ESI^-^ | 53.747 | C_20_H_18_O_6_ | 353.1031 | 353.1026 | 1.31 | 175.0771、227.0712、125.0244 | Gancaonin L/ Licoisoflavanone | GR | F |
| 65 | ESI^+^ | 54.184 | C_15_H_22_O | 219.1743 | 219.1739 | 2.02 | 145.1018、91.0556、77.0385 | Selina-4(14),7(11)-dien-8-one | AM | S |
| 66 | ESI^+^ | 58.499 | C_15_H_20_O_2_ | 233.1536 | 233.1532 | 1.74 | 187.1482、159.1161、145.1010、131.0856、105.0701、91.0538 | Atractylenolide II | AM | S |
| 67 | ESI^+^ | 59.588 | C_20_H_20_O_4_ | 325. 1434 | 325.1428 | 1.95 | 203.1074、189.0911、123.0440 | Glabridin | GR | F |
|  | ESI^-^ | 59.661 |  | 323.1289 | 323.1284 | 1.49 | 201.0921、175.0766、135.0452、213.0919、109.0296 |  |  |  |
| 68 | ESI^-^ | 61.361 | C_20_H_16_O_6_ | 351.0874 | 351.087 | 1.17 | 337.0670 | Licoisoflavone B | GR | F |
| 69 | ESI^-^ | 64.955 | C_25_H_28_O_4_ | 391.1915 | 391.191 | 1.23 | 203.0714、187.1128、159.0821、132.0581 | Kanzonol C or isomer | GR | F |
| 70 | ESI^-^ | 65.756 | C_31_H_46_O_4_ | 481.3323 | 481.3317 | 1.32 | 466.3139、421.3131 | Polyporenic acid C | PR | T |
| 71 | ESI^-^ | 67.048 | C_25_H_28_O_6_ | 423.1813 | 423.1809 | 0.97 | 193.0869、229.0868、174.0313、124.0169 | Gancaonin E or isomer | GR | F |
| 72 | ESI^-^ | 67.745 | C_31_H_50_O_4_ | 485.3636 | 485.363 | 1.31 | 371.2651 | Tumulosic acid | PR | T |
| 73 | ESI^-^ | 68.221 | C_25_H_26_O_6_ | 421.1657 | 421.1652 | 1.1 | 352.0952、309.0400、201.0932、 | Isoangustone A or isomer | GR | F |
| 74 | ESI^-^ | 68.782 | C_25_H_28_O_6_ | 423.1813 | 423.1809 | 0.97 | 193.0873、229.0873、174.0324 | Gancaonin E or isomer | GR | F |
| 75 | ESI^-^ | 68.987 | C_31_H_46_O_5_ | 497.3272 | 497.3266 | 1.3 | 423.2898 | Poricoic acid A(F) | PR | T |
| 76 | ESI^-^ | 69.106 | C_25_H_26_O_6_ | 421.1657 | 421.1652 | 1.1 | 352.0970、309.0423、365.1024 | Isoangustone A or isomer | GR | F |
| 77 | ESI^-^ | 69.208 | C_30_H_46_O_5_ | 485.3272 | 485.3269 | 0.72 | 441.3377、423.3232 | Poricoic acid G | PR | T |
| 78 | ESI^+^ | 69.39 | C_30_H_46_O_4_ | 471.3469 | 471.3458 | 2.3 | 317.2126、189.1636、235.1688 | Glycyrrhetinic acid | GR | T |
|  | ESI^-^ | 69.378 |  | 469.3323 | 469.3318 | 1.14 | 425.3430、355.2655 |  |  |  |
| 79 | ESI^-^ | 73.222 | C_25_H_28_O_4_ | 391.1915 | 391.191 | 1.23 | 203.0706、187.1122、159.0811、132.0670 | Kanzonol C or isomer | GR | F |
| 80 | ESI^-^ | 73.715 | C_25_H_24_O_6_ | 419.15 | 419.1495 | 1.22 | 219.0672、199.0771、133.0567 | Angustone B | GR | F |
| 81 | ESI^-^ | 74.428 | C_30_H_48_O_3_ | 455.3531 | 455.3525 | 1.25 | 136.7823 | Betulinic acid | GR | OA |
| 82 | ESI^-^ | 74.547 | C_33_H_50_O_5_ | 525.3585 | 525.3578 | 1.42 | 59.0147、421.3438、429.8256 | Dehydropachymic acid | PR | T |
| 83 | ESI^-^ | 75.006 | C_33_H_52_O_5_ | 527.3742 | 527.3734 | 1.51 | 465.3364、467.3513 | Pachymic acid | PR | T |

AM, *Atractylodis Macrocephalae Rhizoma*; CR, *Cinnamomi Ramulus*; ESI, electrospray ionization; F, Flavone; G, Glycoside; GR, *Glycyrrhizae Radix et Rhizoma*; OA, Organic acid; P, Phenylpropanoid; PR, *Poria*; S, Sesquiterpene; T, Triterpene.
